# The conifer root rot pathogens *Heterobasidion irregulare* and *Heterobasidion occidentale* employ different strategies to infect Norway spruce

**DOI:** 10.1038/s41598-020-62521-x

**Published:** 2020-04-03

**Authors:** Yang Hu, Malin Elfstrand, Jan Stenlid, Mikael Brandström Durling, Åke Olson

**Affiliations:** 1 Present Address: Zhejiang Academy of Forestry, Liuhe Road, 310023 Hangzhou, China; 2 Department of Forest Mycology and Plant Pathology, Swedish University of Agricultural Sciences, Box 7026, 750 05 Uppsala, Sweden

**Keywords:** Fungal genomics, Fungal pathogenesis

## Abstract

*Heterobasidion irregulare* and *H. occidentale* are two closely related conifer root rot pathogens in the *H. annosum* sensu lato (s.l.) species complex. The two species *H. irregulare* and *H. occidentale* have different host preference with pine and non-pine tree species favored, respectively. The comparison of transcriptomes of *H. irregulare* and *H. occidentale* growing in Norway spruce bark, a susceptible host non-native to North America, showed large differences in gene expression. *Heterobasidion irregulare* induced more genes involved in detoxification of host compounds and in production of secondary metabolites, while the transcriptome induced in *H. occidentale* was more oriented towards carbohydrate degradation. Along with their separated evolutionary history, the difference might be driven by their host preferences as indicated by the differentially expressed genes enriched in particular Gene Ontology terms.

## Introduction

Fungal pathogens use a range of strategies and mechanisms to infect and colonize plants^1^. The degree of specialization vary, from those that infect a wide range of host plants and/or many different tissues, to those that are restricted to a limited number of host species/cultivars or tissues^1–4^. Furthermore, closely related pathogens can utilize different strategies to infect and colonize plants and/or specialize on different hosts^5^. The genetic differences found between two closely related species will be a result of both selection and random genetic drift following reproductive isolation. Although not experimentally proven, differential selection is predicted to have a major impact during speciation in sympatry while both differential selection and random genetic drift is expected to contribute to genetic differences found between species evolving in allopatry. Differences expected to be found in sister taxa could be changes in gene sequences, changes in gene expression, genomic reorganization and gene copy variation^2^.

In plant pathogens, selection has been shown to contribute to the diversification of genes involved in infection, colonization and specialization on host plants, such as genes involved in biosynthesis of secondary metabolites or toxins and genes encoding cell wall degrading enzymes^2,6,7^. Certain effectors, small secreted proteins molecules that facilitating infection and/or triggering defense responses, that participate in determining the host specificity of the closely related oomycete pathogens *Phytophthora infestans* and *P. mirabilis* have been found to be under positive selection^3^. Adaption due to changes in gene expression has been studied to a much lesser degree. In a comparative transcriptome study of the *Brassicaceae* pathogen *Colletotrichum higginsianum* and the cereal pathogen *C. graminicola*, *C. higginsianum* strongly induced secondary metabolism genes while *C. graminicola* did not, suggesting a diversification driven by host interaction^4^.

The basidiomycete *Heterobasidion annosum sensu lato* (s.l.) is a species complex of devastating necrotrophic fungal pathogens that cause root and butt rot to conifers in the northern hemisphere^8,9^. *Heterobasidion annosum* s.l. consist of three European and two North American species with partly overlapping geographic distribution and host preferences^10^. In Europe, the species *H. annosum* s.s. is mostly found attacking Pine spp. but is able to infect other conifers and broad leaved trees as well. In contrast, *H. parviporum* lives almost exclusively on *Picea abies*, but attacks *Abies sibirica* in north-eastern Europe^11,12^, while *H. abietinum* is a pathogen or saprophyte on *Abies* species in southern and central Europe^11,12^. The North American species *H. irregulare* mostly infects *Pinus* and *Juniperus* species but also *Abies balsamea* while *H. occidentale* has a host range excluding *Pinus* spp. but including species in the genera *Abies*, *Picea*, *Pseudotsuga*, *Tsuga, Sequoiadendron*^13^. Although there is a patter in the host preferences among the *H. annosum* s.l. species the specialization has not been driven by co-speciation together with the host^10^. The speciation of *H. annosum* s.l. complex has been suggested to start with a split between an ancestor of the pine infecting species *H. annosum* s.s. and *H. irregulare* and the ancestor of the non-pine infecting species *H. parviporum*, *H. abietinum* and *H. occidentale* in Lauraisa^10^. The common ancestor of *H. annosum* s.s./*H. irregulare* migrated from west Europe to eastern North America. After the Atlantic land bridge disappeared the two species *H. annosum* s.s. and *H. irregulare* evolved in allopatry for several millions of years in Europe and North America, respectively. While the ancestor of *H. parviporum*, *H. abietinum* and *H. occidentale* may originate in east Asia or west North America^10^. In North America the *H. irregulare* continued to spread west over the continent while the spread of *H. occidentale* was restricted to the east by an arid region which probably was colonized by pines, which are a nonhost for *H. occidentale*^10^. After original speciation between pine and non-pine infecting species, *H. irregulare* and *H. occidentale* have had a long period to evolve in allopatry before today’s overlapping geographic distribution in the western North America. How the evolutionary history with speciation in sympatry and a long period of allopatric evolution have shaped their genomes and genetic differences found between *H. irregulare* and *H. occidentale* is not well understood.

Comprehensive studies of *H. irregulare* have revealed a trade off in gene expression between saprotrophic and parasitic lifestyle^14^. During the saprotrophic growth *H. irregulare* use various cellulose and pectin degrading enzymes and many cellulose oxidoreductase genes are up-regulated during wood degradation while genes related to secondary metabolism are more commonly activated during growth in bark^14^. Transcripts identified from *H. annosum* s.s. and Norway spruce interaction showed similar gene induction patterns as seen in *H. irregulare*^15^. Furthermore, a global gene expression study of *H. annosum* s.s. found that the glyoxylate cycle might be important for fungal adaptation to change of nutrients availability in the environment, melanization for adapting to salt stress, and the pathway related to DNA repair for oxidative stress^16^.

We hypothesize that *H. irregulare* and *H. occidentale* evolved distinct genetic machineries for infection and colonization of trees. To analyze the genetic bases for the difference in adaption we performed massive gene expression profiling using deep RNA sequencing of the two pathogens during infection of Norway spruce, a susceptible conifer host not native to North America.

## Results

### Virulence of *H. irregulare* and *H. occidentale* on Norway spruce

Both *H. occidentale* and *H. irregulare* were able to induce necrosis and colonize the sapwood of four-year-old branches of Norway spruce. The success rate of infections was slightly higher for *H. occidentale* than for *H. irregulare* inoculations, 87%, and 73%, respectively. There was no significant difference (One-way ANOVA) between *H. occidentale* and *H. irregulare* when comparing growth in sapwood and expansion of lesions in the inner bark (Table 1). However, for both species there was a significant difference in growth in sapwood and expansion of lesions in the inner bark from two weeks compared to 4 and 6 weeks (Tukey test, P < 0.05).

**Table 1:** Virulence of *H. irregulare* and *H. occidentale* measured as fungal growth in the spruce sapwood, and lesion expansion in the inner bark.

	Growth (mm)			Lesion (mm)		
	2 w	4 w	6 w	2 w	4 w	6 w
*H. occidentale*	16.7 ± 12.6	42.0 ± 28.4	41.0 ± 36.0	7.7 ± 4.2	21.2 ± 16.6	28.2 ± 11.0
*H. irregulare*	23.8 ± 11.1	58.3 ± 7.6	62.5 ± 20.6	9.5 ± 7.6	11.0 ± 5.0	20.5 ± 7.2

Growth = Fungal growth in the sapwood, Lesion = lesion length in the inner bark, 2 w, 4 w and 6 w = 2, 4 and 6 weeks after inoculation (n = 3–5)^a^.

^a^No significant difference between *H. occidentale* and *H. irregulare* (One-way ANOVA, P > 0.5) but a significant difference in growth in sapwood and expansion of lesions in the inner bark from two weeks compared to 4 and 6 weeks (Tukey test, P < 0.05).

### Genome annotation and gene orthologue identification

The genome sequences of *H. irregulare* and *H. occidentale* were acquired from previous published data^14,17^. By re-annotating the genomes using MAKER, 9462 gene models were identified in *H. irregulare* and 10295 in *H. occidentale*. Although, we identified 2002 fewer gene models with the MAKER annotation than the published version of the *H. irregulare* genome the majority 8412 (89%) gene models were shared with the *H. irregulare* genome available at Joint Genome Institute (JGI) website (http://genome.jgi.doe.gov/Hetan2/Hetan2.home.html). The two species, *H. irregulare* and *H. occidentale* share 7545 one-to-one orthologous gene models. The gene models of the two species could be further grouped together into 8306 orthoMCL clusters.

### Genes induced in *H. irregulare* and *H. occidentale* during infection

**Figure 1:**
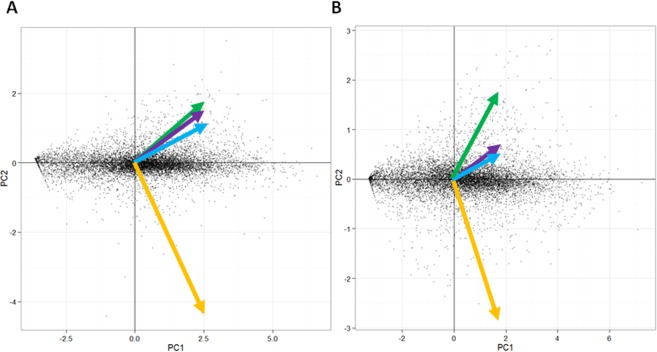
Principle component analysis (PCA) of significantly differentially expressed genes of *H. irregulare* (**A**) and *H. occidentale* (**B**). Arrows indicated the directions of components: orange arrow represented fungus grow in liquid culture, green, blue, purple arrows represented 2, 4, and 6 weeks after infection.

In total 179 GB RNA sequence data from 2, 4 and 6 weeks (2 w, 4 w and 6 w) of infected bark and liquid (L) culture control samples were generated by the Illumina Hiseq (European Nucleotide Archive with accession number: PRJEB33431). After length and quality filtering 30.5 to 154.8 million reads per bark sample and 10 to14 million reads per pure fungal sample were retained (Table 2). The sequence reads from the samples were mapped against their respective reference genome. From the pure fungal samples, 77 to 84% of the read pairs mapped, while from the bark samples 0.4 to 21.9% of the read pairs mapped (Table 2). The numbers of mapped pairs from the colonized bark samples were more than 500 000 except for one replicate of one sample which generated 229 570 mapped pairs (Table 2). The assembly of mapped reads identified 9491 gene models in *H. irregulare* which is 17 more than the gene models annotated in the genome by MAKER while in *H. occidentale* 10220 gene models were identified which are 75 less than the MAKER annotation of the genome. A PCA analysis of the expression fragments per kilobase of exon per million mapped reads (FPKM) of all genes in the four conditions for each species suggested that, the L samples are substantially different from the interaction samples taken from bark (Fig. 1).

**Table 2:** Total number of sequence reads generated and aligned sequence pairs from *H. irregulare* and *H. occidentale* liquid cultures and 2, 4, and 6 weeks infected Norway spruce bark.

Samples names*	Total reads	Aligned pairs	Mapped pairs (%)
TC32-1-L-rep1	11809378	9114879	77.2
TC32-1-L-rep2	10555531	8764947	83.0
TC32-1-L-rep3	13240830	11124807	84.0
TC32-1-2w-rep1	39748040	1148233	2.9
TC32-1-2w-rep2	43180655	1637664	3.8
TC32-1-2w-rep3	40750502	1282462	3.1
TC32-1-4w-rep1	121996688	583954	0.5
TC32-1-4w-rep2	49434129	1241663	2.5
TC32-1-4w-rep3	95247997	3219921	3.4
TC32-1-6w-rep1	58357874	229570	0.4
TC32-1-6w-rep2	53266103	681649	1.3
TC32-1-6w-rep3	55675183	975852	1.8
TC122-12-L-rep1	13661132	11135225	81.5
TC122-12-L-rep2	12389419	10207752	82.4
TC122-12-L-rep3	11098200	9092986	81.9
TC122-12-2w-rep1	30863928	6764247	21.9
TC122-12-2w-rep2	39222132	1928245	4.9
TC122-12-2w-rep3	33431531	2346657	7.0
TC122-12-4w-rep1	154786107	1519679	1.0
TC122-12-4w-rep2	78321161	1714337	2.2
TC122-12-4w-rep3	32287019	4234747	13.1
TC122-12-6w-rep1	30472557	5817262	19.1
TC122-12-6w-rep2	73805957	3078694	4.2
TC122-12-6w-rep3	142671388	3277956	2.3

*H. irregulare* = TC32-1 and *H. occidentale* = TC122-12; L = liquid culture, 2 w, 4 w and 6 w = 2, 4, and 6 weeks after inoculating Norway spruce bark with the fungus; rep1, rep2 and rep3 = replicate 1, 2, and 3.

**Figure 2:**
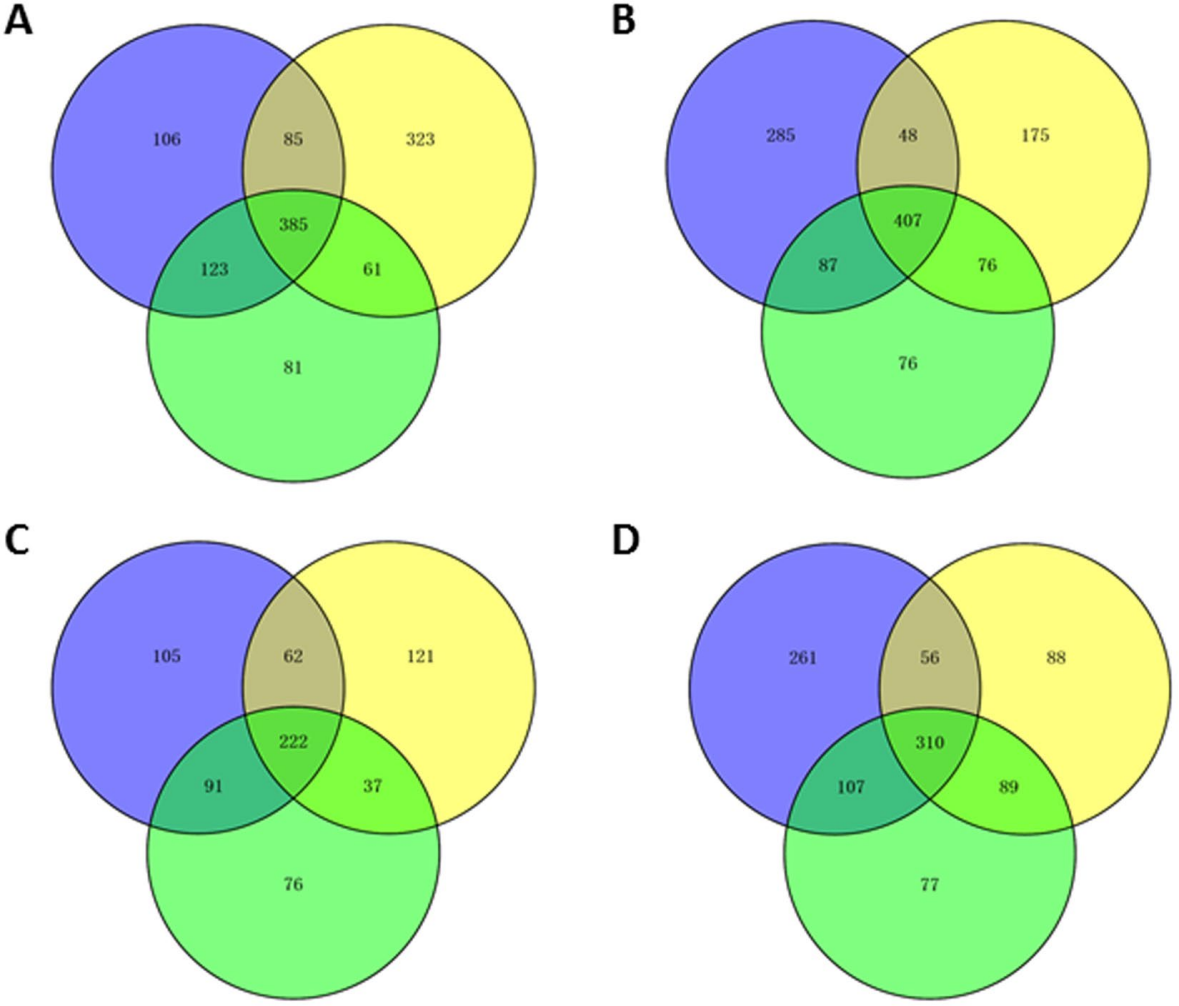
Venn diagram comparing the number of significantly differentially up- or down-regulated genes during infection of Norway spruce in *H. irregulare* or *H. occidentale*. The purple, green and pink represent the number of significant up- or down-regulated genes from 2, 4 and 6 weeks after infection compare to liquid culture. The number of common and different up-regulated genes at 2, 4, and 6 weeks after inoculation compared with liquid culture of *H. irregulare* (**A**). The number of common and different up-regulated genes at 2, 4, and 6 weeks after inoculation compared with liquid culture of *H. occidentale* (**B**). The number of common and different down-regulated genes at 2, 4, and 6 weeks after inoculation compared with liquid culture of *H. irregulare* (**C**). The number of common and different down-regulated genes at 2, 4. and 6 weeks after inoculation compared with liquid culture of *H. occidentale* (**D**).

The total number of significant differentially expressed genes (DEGs) between any two of the treatments: pure fungal culture (L) and fungal colonization of Norway spruce bark (2 w, 4 w and 6 w) of *H. irregulare* and *H. occidentale* were 2081 and 2360, respectively. The number of DEGs in any of the three time points in bark compared to L were between 1076 and 1561 while the number of DEGs between the three time points in bark was much lower for both species (Table S1). In *H. irregulare* there are 385 genes consistently up-regulated and 222 consistently down-regulated genes in 2 w, 4 w and 6 w compared to L (Fig. 2A,C). Similarly, 407 and 310 genes were consistently up- and down-regulated, respectively, in *H. occidentale* when comparing infection with liquid culture control (Fig. 2B,D).

### Annotation and enrichment test of DEGs from *H. irregulare* and *H. occidentale*

Gene ontology (GO) annotation were provided for 5685 out of 9474 gene models for *H. irregulare* and 5759 out of 10295 gene models for *H. occidentale*. There were enrichment of genes in 11, 12 and 15 categories for biological processes among the DEGs up-regulated for *H. irregulare* in 2 w, 4 w and 6 w and 13, 6 and 6 categories for *H. occidentale* (Fisher’s exact test with a FDR threshold of 5%) (Table 3, S2 and S3). Genes associated with oxidation-reduction process (GO:0055114), transmembrane transport (GO:0055085), amino acid transmembrane transport (GO:0003333) and small molecule catabolic process (GO:0044282) are consistently up-regulated and enriched in both species (Table S2). Among the genes found in categories uniquely enriched in *H. irregulare*, and that were consistently up-regulated during infection, several were related to detoxification, such as alpha-amino acid catabolic process (GO:1901606), drug transport (GO:0015893), benzoate metabolic process (GO:0018874) and xenobiotic catabolic process (GO:0042178). In contrast, genes associated with carbohydrate metabolism and transport were uniquely enriched in *H. occidentale*, especially genes in the categories carbohydrate transport (GO:0008643), polysaccharide metabolic process (GO:0005976), cellular carbohydrate metabolic process (GO:0044262) (Table S2).

**Table 3:** The numbers of GO terms enriched in *H. irregulare* and *H. occidentale*.

GO categories	*H. irregulare*				*H. occidentale*				Shared
	2 w	4 w	6 w	Consistent	2 w	4 w	6 w	Consistent	Consistent
Cellular Localization	2	3	1	1	2	0	2	0	0
Molecular Function	9	13	10	8	15	9	9	9	3
Biological Process	11	12	15	8	13	6	6	8	4

The down-regulated DEGs are associated with a smaller number of GO categories (Table S3). In *H. irregulare*, the down-regulated DEGs are related to fungal cell wall biogenesis and certain type of carbohydrate metabolism, such as fungal-type cell wall (GO:0009277) and serine-type carboxypeptidase activity (GO:0004185). In *H. occidentale*, the down-regulated DEGs are more diverse and includes both nutrient metabolism related to growth like amino sugar metabolic process (GO:0006040) and flavin adenine dinucleotide binding (GO:0050660). Although genes in the categories GO:0050660 and GO:0016614 are enriched in the up-regulated genes of *H. irregulare* and enriched among the down-regulated DEGs in *H. occidentale* the actual genes under these GO categories are not orthologous genes.

### Genes commonly expressed for *H. irregulare* and *H. occidentale* during infection

**Figure 3:**
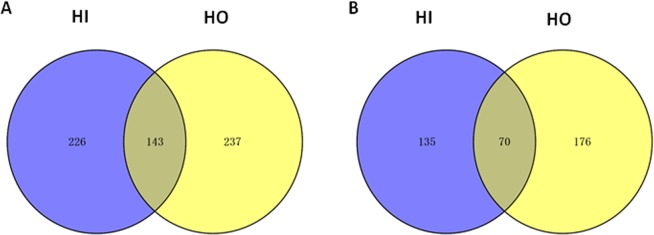
The number of common and different consistently up-regulated (**A**) and down-regulated (**A**) genes of *H. irregulare* and *H. occidentale* (HI = *H. irregulare*, HO = *H. occidentale*).

Among the consistently, over the three time point, up-regulated DEGs, 369 in *H. irregulare* and 380 in *H. occidentale* share an orthologue in the other genome. However, only 143 gene models with orthologues are consistently up-regulated in bark in the two species (Fig. 3, Table S4). Among the consistently down-regulated genes in bark there were 70 orthologous gene pairs (Fig. 3, Table S5). Additionally, nine *H. irregulare* genes correspond to 11 *H. occidentale* genes which do not have reciprocal orthologues but are in the same gene families, were also among the shared up-regulated genes. Among the 143 shared DEGs up-regulated in bark, a number of host material degradation enzymes, transmembrane transporters, and genes involved in metabolism are identified (Table S4).

### Differently expressed genes in *H. irregulare* and *H. occidentale* in bark

**Figure 4:**
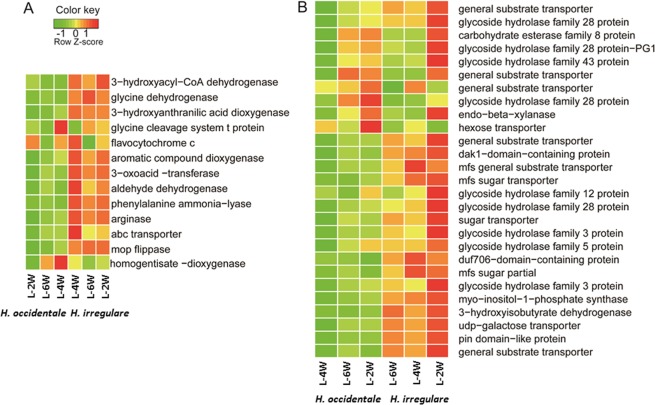
Differentially regulated genes within the enriched GO catalogue of *H. irregulare* (alpha-amino acid process, GO:1901606; drug transport, GO:0015893 and benzoate metabolic process, GO:0018874) and *H. occidentale* (carbohydrate transport, GO:0008643; polysaccharide metabolic process, GO:0005976; and cellular carbohydrate metabolic process, GO:0044262) as well as their corresponding genes from another species. The heat map of genes expression of log2 fold change from *H. irregulare* of enriched GO terms comparing with their orthologous in *H. occidentale* (**A**) and *H. occidentale* enriched GO terms comparing with their orthologous in *H. irregulare* (**B**).

Out of the genes specifically differentially up-regulated in *H. irregulare* and *H. occidentale* 226 and 237 have orthologues in the other species were the gene was not up-regulated. In addition, there were 15 *H. irregulare* and 22 *H. occidentale* genes specifically induced for which we could not identify an orthologue in the other species. Consequently, a set of 241 *H. irregulare* gene and 259 *H. occidentale* genes corresponding to two third of the consistently up-regulated DEGs were differentially regulated between the two species, and were used for further analysis. In total 91 of 241 *H. irregulare* genes were assigned Kyoto Encyclopedia of Genes and Genomes (KEGG) Orthology terms and were mapped to 102 KEGG pathways, as well as 87 of 259 *H. occidentale* genes that were mapped to 85 KEGG pathways. Approximately half of the KEGG pathways identified that contains species specifically differentially regulated genes in *H. irregulare* and *H. occidentale* were unique to either species (Table S8). The genes assigned to the enriched GO terms in *H. irregulare* (alpha-amino acid process, GO:1901606; drug transport, GO:0015893 and benzoate metabolic process, GO:0018874) and *H. occidentale* (carbohydrate transport, GO:0008643; polysaccharide metabolic process, GO:0005976; and cellular carbohydrate metabolic process, GO:0044262) show different expression patterns compared to orthologues in the other species(Fig. 4A,B).

## Discussion

The closely related species *H. irregulare* and *H. occidentale*, in the species complex *H. annosum* s.l., showed surprisingly small difference in gene content, a majority of genes could be found as one-to-one orthologous or in groups of gene families. However, the species show large differences in gene expression patterns *in planta* when inoculated on the same host species (which is not the natural host for either species). Approximately 2/3 of the genes consistently differentially expressed during growth in bark in either species are specifically induced in that species. The difference in enriched GO terms of gene expression in spruce bark between *H. irregulare* and *H. occidentale* indicate that the differences found are likely to be a result of adaption to their particular infection strategy. If the changes in gene expression between two species are a result of a random process or a result of stabilizing selection, the expression pattern would show random variation or little variation rather than variation of genes enriched in certain orthology groups. A previous study of the evolutionary history of *H. annosum* s.l. shows that separation of the *H. irregulare* and *H. occidentale* common ancestor to modern species is associated with the host preference of pine infection and non-pine infection^10^. Thus the difference in gene expression patterns between pine infecting *H. irregulare* and non-pine infecting *H. occidentale* most likely is associated with this hallmark trait in the evolutionary history of these taxa.

The comparisons of the transcriptomes of *H. irregulare* and *H. occidentale* growing in Norway spruce bark give insights into the different strategies the two species have for infecting their respective main host tree species. The transcriptional patterns shown by *H. irregulare* accentuate tolerance to the harsh environment created by host defense and production of secondary metabolites for attacking its host (Fig. 4A). When fungal pathogen invasion is detected by plants, the plant mounts a defense that among other mechanisms, involve production of specialized toxic metabolites. Those chemicals are often terpenes, phenolics and nitrogen containing compounds^18^. To successfully colonize their host, pathogens have to overcome the antimicrobial effects of such chemicals by employing their xenobiotic metabolizing enzymes such as cytochrome P450s or glucosyltransferases^19,20^. We show that both *H. irregulare* and *H. occidentale* induce expression of the nitrogen compound metabolism, benzoate metabolic and xenobiotic catabolic enzymes as well as the multidrug transporters to detoxify host defense chemicals to grow in spruce bark. However, the transcriptome of *H. irregulare* showed induction of more genes involved in detoxification than did *H. occidentale*, GO terms related to detoxification were only found to be enriched in *H. irregulare’s* interaction with the Norway spruce. An aromatic compound dioxygenase is particularly up-regulated in *H. irregulare*. Aromatic compound dioxygenase catalyzes the oxidative ring cleavage of catechol and might be involved in detoxification of phenolic compounds produced by the host. In *H. annosum* s.s. infected tissues, stilbenes converted to ring-opened, de-glycosylated, and dimeric products are found^21^. Furthermore, analyses of *Endoconidiophora polonica* protein and metabolite extracts have shown that these stilbene metabolites arise from fungal enzyme activities^22^ and that *E. polonica* most likely use these small metabolites as carbon sources for growth^23^. Possibly, *H. irregulare* uses the aromatic compound dioxygenase to generate linearized stilbene metabolites for its nutrition. To avoid the effects of antimicrobial compounds, effluxing them out of the fungal cells is another important mechanism for handling them.

Production of low-molecular weight toxins is proposed to be an important virulence factor of many necrotrophic plant pathogens and *H. annosum* s.l. is known to produce toxins such as, oosponol, fomannosin, fomannoxin, and the fomjorins^24^. However, secondary metabolite profiling has showed that there is a lager difference in compound composition between the pine infecting species and the non-pine infecting species than with in each of the groups^25^. Here we show that differential up-regulated enzymatic genes from the two species to a large extent mapped to different KEGG pathways related to secondary metabolism. This result suggests that compounds produced during infection were different in *H. irregulare* and *H. occidentale*.

Interestingly, we also found a differential shift of primary metabolism in *H. irregulare* during infection. The glyoxylate cycle is only induced in *H. irregulare*, which is similar to what has been reported for *Fusarium graminearum* growing in wheat^26^. Two key enzymes, an isocitrate lyase and a malate synthase, in the glyoxylate cycle were highly induced and with high expression level. The glyoxylate cycle requires a mitochondrial inner membrane carriers to transport isocitrate to the cytosol^26^. Mitochondrial carrier genes have also been shown to be important for virulence of *Candida albicans*^27^. The glyoxylate cycle could be important for *H. irregulare* growing in host and it could also be related to previous observation of mitochondrial involvement in *H. annosum* s.l. virulence^28^. In addition, genes of the glyoxylate cycle have been found up-regulated in *H. annosum* s.s. when facing nutrient starvation and they might be important for survival of the fungus during stress conditions^26,27,29^. There are three mitochondrial carriers and one mitochondrial inner membrane carrier up-regulated in *H. irregulare* during infection. Knock out one of mitochondrial carrier gene (*CIC1*) or (*FOW1*) in *F. graminearum* or *F. oxysporum* to disrupt the glyoxylase cycle, resulted in mutants that performed well *in vitro* but had a 2/3 reduced lesion size in infected coleoptiles^26,29^. This suggest that as in many plant pathogens mitochondrial function is important for successful colonization of hosts.

Several quantitative trait loci (QTL) for virulence of *H. irregulare* have been identified^30^. By re-mapping the virulence data from Lind *et al*.^30^, 619 gene models have been annotated in the QTL regions^14^. With our updated annotation, there are 389 gene models still found in the QTL regions. Totally, 13 genes in the QTL regions have been shown to be consistently significantly up-regulated and 10 down-regulated. The up-regulated genes included three transporters and two transferases which further supported our previous conclusions that detoxification of anti-fungal substrates from host resistance responses, is important for the virulence of *H. irregulare*. A gene mentioned by Olson *et al*.^14^, the putative Flavin containing Baeyer-Villiger monooxygenase (JGI proteinID 58532) was found up-regulated around four times at two weeks after inoculation. The product of this gene is potentially involved in the biosynthesis of the toxin fomannosin, which is an important virulence factor in *H. annosum* s.l.^31^.

Compared to *H. irregulare*, *H. occidentale* appears to use more cell wall degrading enzymes (CWDEs) during infection, possibly to utilize the local nutrient source more efficiently and exploit the substrate more intensively than *H. irregulare* (Fig. 4B). The emphasis on cell wall degradation in the *H. occidentale* transcriptome could be interpreted as *H. occidentale* invests more in biomass production than *H. irregulare*, a difference in colonization strategy that might partly explain why we found higher proportion of fungal RNA sequence reads in *H. occidentale* infected bark samples than in *H. irregulare* infected bark samples. Although there were genes of glycoside hydrolyze (GH) families significantly up-regulated in both in *H. irregulare* and *H. occidentale*, many more of them were highly expressed in *H. occidentale* than in *H. irregulare*, especially at two weeks after the infection. This indicates that *H. occidentale* employ those enzymes to degrade the host cell structure at earlier infection stage for use as nutrient and carbon source. This type of virulence behavior has been found in other necrotrophic pathogens^2^. There is some evidence that the GH28 family of CWDEs act as virulence factors in *B. cinerea*^32^, *Alternaria citri*^33^ and *Aspergillus flavus*^34^ where they are involved in cell wall decomposition and tissue maceration. Importantly, a *Claviceps purpurea* strain carrying a deletion of two GH28 genes renders the fungus nearly non-pathogenic on rye without affecting its vegetative growth properties^35^. Here, one GH28 was found up-regulated in both *H. irregulare* and *H. occidentale*, and three additional GH28s and a number of other GHs for degrading cellulose and hemi-cellulose showed higher induction levels in *H. occidentale* than in *H. irregulare*, which indicate that GH28 might play an important role for *H. occidentale* to survive and spread throughout woody tissues during infection of Norway spruce. Hu *et al*.^36^ characterized one GH28 gene encoding endo-rhamnogalacturonase (*HIRHG*) located in the virulence QTLs of *H. irregulare*^14,30^. They found that *HIRHG* was up-regulated during infection and maybe important for the pathogen to grow in a pectin containing environment. Liu *et al*.^37^ compared the transcriptomes of two different *H. occidentale* isolates grown on apple fruits. Their results suggest that the capacity of *H. occidentale* to colonize apple fruit correlated with the expression of potential carbohydrate active enzyme genes^37^.

Even though there are marked differences in consistently up-regulated genes between *H. irregulare* and *H. occidentale* during infection, there are still common features for the pathogenicity. There are large numbers of transporters up-regulated in both species. Transporters are important for pathogens to acquire nutrients from host tissue and efflux of toxic compounds. One ABC transporter belonging to subgroup G (ABC-G) was highly induced in both *H. irregulare* and *H. occidentale*, but with higher fold change in *H. irregulare*. ABC transporters have been showed important for tolerance to phytoalexins and to achieve full virulence in many fungal plant pathogens, such as, *Fusarium sambucinum*, *Mycosphaerella graminicola* and *B. cinerea*^38–40^. The ABC-Gs involved in efflux of toxic compounds are referred to as multidrug resistance-associated proteins. Especially in the fungal-fungal interaction of the mycoparasite *Clonostachys rosea*, the ABC-Gs are highly induced by particular mycotoxin and ABC-G5 have been confirmed important for xenobiotic tolerance by gene disruption and complementation^41,42^. The activity of aromatic compound biosynthetic processes is possibly related to production of toxins. Similarly, a clavaminate synthase-like protein belonging to the alpha-ketoglutarae-dependent oxygenases was highly expressed. This enzyme has been observed in interaction between *H. annosum* s.s and Norway spruce, and was predicted be involved in secondary metabolism for fungal toxin productions^15^.

In general, the pine infecting species, *H. annosum* s.s. and *H. irregulare* are more effective than the other species within the species complex in colonizing the cambial layer and sapwood of their hosts, both in the root system and at or just above the root collar^43^. In comparison, infection by non-pine infecting species, such as *H. parviporum*, is normally confined to the heart wood in the vast majority of root rot infected Norway spruce in Europe^12^. Since a trade-off between saprotrophic decomposition and necrotrophic parasitism in *H. irregulare* have been suggested^14^ it is tempting to suggest that the transcriptional differences found between *H. irregulare* and *H. occidentale* could indicate that *H. irregulare* might be more prone to use necrotrophic parasitism in interactions with a host compared to *H. occidentale* that mainly grow in the heart wood of trees and rely on its saprobic capacity to invade its host.

## Material and Methods

### Fungal isolates, plants and inoculation method

The *H. irregulare* strain TC32-1 and *H. occidentale* strain TC122-12 were used in this study and maintained on Hagem agar (HA) medium^9^ at 4 °C for storage, and 20 °C for growth. For inoculation of Norway spruce, pieces of spruce wood (∅5 × 5 mm) were placed on one-week-old mycelia grown on HA medium. The plates were placed at 20 °C in darkness, and incubated for four weeks to allow the fungi to colonize the wood pieces.

Six trees were used for inoculations, three ramets from two progeny lines each, of the age of eight years, form the Norway spruce family S21H9820005^44^. Prior to inoculation, the trees were grown for one month in a greenhouse at 20–22 °C temperature at day time, 16–20 °C at night time with 16 hours’ light. Nine branches of the age of four years were selected from each tree for inoculation. Each branch was inoculated at three points with *H. irregulare* strain TC32–1 and *H. occidentale* strain TC122-12 and one isolate not included in this study. At each inoculation site, a round piece of ∅5 mm tree bark tissue was removed and colonized wood dowels inserted into the wound and attached by wrapping tightly with Parafilm® according to previous established method^45^. The distances between inoculation sites within the same branch were kept to a minimum of 15 cm to avoid cross contamination. Three branches per tree was harvested at each time point and the bark samples from two sites closest to the main stems were taken for RNA extraction and the remaining tissue was used for virulence test after two, four and six weeks. The virulence tests of *H. irregulare* and *H. occidentale* were scored according established methods^45^.

### RNA extraction and sequencing

Strains of *H. irregulare* and *H. occidentale* grown in liquid HA for two weeks and spruce bark infected by *H. annosum* s.l. for 2, 4 and 6 weeks as mentioned previously were harvested for RNA isolation. The total RNA was isolated as described by Chang *et al*.^46^ and stored at −80 °C until used. The RNA 6000 Nano Kit (Agilent Technologies) was used to evaluate quantity and integrity of total RNA with Bio-analyzer 2001. Total RNA was treated with DNase I (Sigma-Aldrich) to eliminate contamination of genomic DNA. Library construction and cDNA synthesis was performed at the SNP&SEQ Technology Platform of Uppsala University Hospital. High-throughput sequencing was also performed at the same place using the Illumina Hiseq (Illumina, San Diego, CA, USA) according to standard protocols. The samples were sequenced for ‘paired-end’ reads across one Illumina lane.

### RNAseq data analysis

Nesoni (https://github.com/Victorian-Bioinformatics-Consortium/nesoni) was used to filter the Illumina reads based on a phred-scale quality score cut off of 20 and reads length cut off 50 basepairs. All the filtered reads from the *H. irregulare* and *H. occidentale* samples were mapped to their own genomes by Tophat2^47^. The reads were mapped to their respective genome without any reads mismatches, and adjusted the mate-inner- distance to 20, min-intron-length, min-coverage-intron and min-segment-intron to 5, max-intron-length, max-coverage-intron and max-segment-intron to 5000, and read-realign-edit-dist to 0, and set library type to fr-firststrand. Differential expression analysis was performed with the software package Cufflinks (2.1.1) to quantify transcript abundance in terms of fragments per kilobase of exon per million mapped reads (FPKM). The transcriptomes were assembled in each mapped alignment strictly according the reference genome annotation, using activation of the multi-read-correct and setting the overlap-radius to 1, intron and library parameters as well as in Tophat2. Further, the individual assemblies were merged to a final annotation for each species by cuffmerge and quantification of the gene and transcript expression were done using cuffquant. The differentially expressed genes (DEGs) were finally identified by cuffdiff based on q-value (false discovery rate (FDR))  <  0.05 and visualized using CummeRbund.

### Annotation, Gene Ontology (GO) enrichment test and identification of gene orthologous between *H. irregulare* and *H. occidentale*

In order to obtain comparable gene sets from the two species, the genome sequences of *H. irregulare* and *H. occidentale* acquired from previous published data^14,17^ were both annotated using the MAKER2 genome annotation pipeline (version 2.31.8)^48^. Maker was configured to use the Augustus, SNAP and GeneMarkES ab initio gene predictors. To support the annotations, RNASeq data from the liquid cultures was assembled using Trinity^49^ and provided to MAKER. Orthology of gene models between the two studies species was established by determining the best reciprocal blast matches between the two sets of predicted transcripts.

BLAST2GO v3.10^50^ was used to improve the annotation of *H. irregulare* and *H. occidentale* transcripts as well as to assess GO term enrichment. Functional categories were assigned to the differentially expressed genes according to the GO system using Blast2GO, enabling the integrated Interproscan and ANNEX functions for further improvement of annotations. Enrichment of GO terms in biological process, molecular function, and cellular component categories of genes significantly up- and down-regulated in 2w, 4w and 6w, respectively, compared to liquid culture of each species were evaluated by comparing to the gene models of each genome using Fisher’s exact test with a FDR threshold of 5%. In addition, protein sequence were submitted in KAAS (KEGG automatic annotation server) and KEGG orthology (KO) assignments^51^ were obtained based on bi-directional best-hit of BLAST searching to a threshold of 60 with the GENES data set of KAAS defaulted Eukaryotes plus *Neurospora crassa, Magnaporthe oryzae, Fusarium graminearum, Sclerotinia sclerotiorum, Botrytis cinerea, Aspergillus nidulans, Aspergillus fumigatus, Aspergillus oryzae, Aspergillus niger, Phaeosphaeria nodorum, Cryptococcus neoformans* JEC21, *Postia placenta* and *Ustilago maydis*^52^.

### Statistical analysis

Phenotypic data were analyzed by analysis of variance (ANOVA) using a general linear model implemented in using Minitab Statistical software 16 (Minitab Inc., State Collage, PA). Pairwise comparisons were made using Tukey´s test at the 95% significant level.

## Supplementary information

Supplementary Tables S1-S8.
