# Supplementary material for: The conifer root rot pathogens Heterobasidion irregulare and Heterobasidion occidentale employ different strategies to infect Norway spruce
Source: Sci Rep. 2020 Apr 3;10:5884. doi: 10.1038/s41598-020-62521-x (PMC7125170; doi:10.1038/s41598-020-62521-x)
Supplement: Supplementary file 1 — Supplementary Tables S1-S8. [file 41598_2020_62521_MOESM1_ESM.docx]

**Supplementary Information**

**The conifer root rot pathogens *Heterobasidion irregulare* and *Heterobasidion occidentale* employ different strategies to infect Norway spruce**

Yang Hu^1^, Malin Elfstrand^2^, Jan Stenlid^2^, Mikael Brandström Durling^2^ and Åke Olson^2*^

^1^ Zhejiang Academy of Forestry, Liuhe Road,310023 Hangzhou, China

^2^ Department of Forest Mycology and Plant Pathology, Swedish University of Agricultural Sciences, Box 7026, 750 05 Uppsala, Sweden

* Corresponding author

Table s1. The numbers of genes significantly differential expressed

| species | sample 1 | sample 2 | genes | Diff(q=0.05) | up | down |
| --- | --- | --- | --- | --- | --- | --- |
| *H. irregulare* | Liquid | 2weeks | 9491 | 1179 | 699 | 480 |
|  | Liquid | 4weeks | 9491 | 1296 | 854 | 442 |
|  | Liquid | 6weeks | 9491 | 1076 | 651 | 425 |
|  | 2weeks | 4weeks | 9491 | 603 | 372 | 231 |
|  | 2weeks | 6weeks | 9491 | 229 | 135 | 94 |
|  | 4weeks | 6weeks | 9491 | 531 | 185 | 346 |
| *H. occidentale* | Liquid | 2weeks | 10220 | 1561 | 827 | 734 |
|  | Liquid | 4weeks | 10220 | 1249 | 706 | 543 |
|  | Liquid | 6weeks | 10220 | 1229 | 646 | 583 |
|  | 2weeks | 4weeks | 10220 | 797 | 452 | 345 |
|  | 2weeks | 6weeks | 10220 | 576 | 262 | 314 |
|  | 4weeks | 6weeks | 10220 | 200 | 75 | 125 |

Table s2 The enriched GO terms of up-regulated genes in liquid culture compare to 2, 4 and 6weeks after infection and the consistent up-regulated genes in bark. l-2(4,6,c)= liquid culture compare to 2-,4- and 6- weeks and CUGs, C= Cellular localization, P= Biological Process and F=Molecular Function

|  | *H. irregulare* | |  |  | *H. occidentale* | | |  |
| --- | --- | --- | --- | --- | --- | --- | --- | --- |
|  | GO-ID | Term | Category | No. of genes | GO-ID | Term | Category | No. of genes |
| l-2 | GO:0016021 | integral component of membrane | C | 95 | GO:0005576 | extracellular region | C | 20 |
|  | GO:0005576 | extracellular region | C | 14 | GO:0042555 | MCM complex | C | 4 |
|  | GO:0015171 | amino acid transmembrane transporter activity | F | 12 | GO:0030248 | cellulose binding | F | 13 |
|  | GO:0050660 | flavin adenine dinucleotide binding | F | 28 | GO:0003995 | acyl-CoA dehydrogenase activity | F | 9 |
|  | GO:0030248 | cellulose binding | F | 9 | GO:0015925 | galactosidase activity | F | 8 |
|  | GO:0016614 | oxidoreductase activity, acting on CH-OH group of donors | F | 25 | GO:0004497 | monooxygenase activity | F | 34 |
|  | GO:0051213 | dioxygenase activity | F | 13 | GO:0020037 | heme binding | F | 39 |
|  | GO:0016620 | oxidoreductase activity, acting on the aldehyde or oxo group of donors, NAD or NADP as acceptor | F | 9 | GO:0016705 | oxidoreductase activity, acting on paired donors, with incorporation or reduction of molecular oxygen | F | 41 |
|  | GO:0016701 | oxidoreductase activity, acting on single donors with incorporation of molecular oxygen | F | 7 | GO:0005506 | iron ion binding | F | 38 |
|  | GO:0004650 | polygalacturonase activity | F | 5 | GO:0004650 | polygalacturonase activity | F | 5 |
|  | GO:0010181 | FMN binding | F | 10 | GO:0051213 | dioxygenase activity | F | 13 |
|  | GO:0055114 | oxidation-reduction process | P | 149 | GO:0008810 | cellulase activity | F | 5 |
|  | GO:0003333 | amino acid transmembrane transport | P | 12 | GO:0030599 | pectinesterase activity | F | 3 |
|  | GO:0071555 | cell wall organization | P | 6 | GO:0097599 | xylanase activity | F | 3 |
|  | GO:1901606 | alpha-amino acid catabolic process | P | 13 | GO:0052692 | raffinose alpha-galactosidase activity | F | 3 |
|  | GO:0008643 | carbohydrate transport | P | 14 | GO:0045330 | aspartyl esterase activity | F | 3 |
|  | GO:0042537 | benzene-containing compound metabolic process | P | 7 | GO:0008509 | anion transmembrane transporter activity | F | 11 |
|  | GO:0006073 | cellular glucan metabolic process | P | 13 | GO:0044699 | single-organism process | P | 314 |
|  | GO:0019318 | hexose metabolic process | P | 14 | GO:0055114 | oxidation-reduction process | P | 135 |
|  | GO:0042493 | response to drug | P | 4 | GO:0044262 | cellular carbohydrate metabolic process | P | 27 |
|  | GO:0000272 | polysaccharide catabolic process | P | 8 | GO:0000272 | polysaccharide catabolic process | P | 13 |
|  | GO:0046487 | glyoxylate metabolic process | P | 6 | GO:0055085 | transmembrane transport | P | 70 |
|  |  |  |  |  | GO:0005985 | sucrose metabolic process | P | 16 |
|  |  |  |  |  | GO:0005982 | starch metabolic process | P | 16 |
|  |  |  |  |  | GO:0008643 | carbohydrate transport | P | 14 |
|  |  |  |  |  | GO:0071103 | DNA conformation change | P | 13 |
|  |  |  |  |  | GO:0032508 | DNA duplex unwinding | P | 8 |
|  |  |  |  |  | GO:0070887 | cellular response to chemical stimulus | P | 9 |
|  |  |  |  |  | GO:0042545 | cell wall modification | P | 3 |
|  |  |  |  |  | GO:0006260 | DNA replication | P | 14 |
| l-4 | GO:0070069 | cytochrome complex | C | 6 | GO:0004553 | hydrolase activity, hydrolyzing O-glycosyl compounds | F | 34 |
|  | GO:0016021 | integral component of membrane | C | 112 | GO:0005506 | iron ion binding | F | 38 |
|  | GO:0098803 | respiratory chain complex | C | 8 | GO:0016705 | oxidoreductase activity, acting on paired donors, with incorporation or reduction of molecular oxygen | F | 37 |
|  | GO:0015171 | amino acid transmembrane transporter activity | F | 12 | GO:0051213 | dioxygenase activity | F | 14 |
|  | GO:0005507 | copper ion binding | F | 9 | GO:0004497 | monooxygenase activity | F | 29 |
|  | GO:0051213 | dioxygenase activity | F | 13 | GO:0015171 | amino acid transmembrane transporter activity | F | 8 |
|  | GO:0009055 | electron carrier activity | F | 10 | GO:0020037 | heme binding | F | 32 |
|  | GO:0071949 | FAD binding | F | 11 | GO:0003995 | acyl-CoA dehydrogenase activity | F | 5 |
|  | GO:0010181 | FMN binding | F | 10 | GO:0016701 | oxidoreductase activity, acting on single donors with incorporation of molecular oxygen | F | 6 |
|  | GO:0020037 | heme binding | F | 32 | GO:0055114 | oxidation-reduction process | P | 121 |
|  | GO:0005506 | iron ion binding | F | 35 | GO:0055085 | transmembrane transport | P | 61 |
|  | GO:0008374 | O-acyltransferase activity | F | 5 | GO:0003333 | amino acid transmembrane transport | P | 8 |
|  | GO:0016705 | oxidoreductase activity, acting on paired donors, with incorporation or reduction of molecular oxygen | F | 33 | GO:0008643 | carbohydrate transport | P | 11 |
|  | GO:0016701 | oxidoreductase activity, acting on single donors with incorporation of molecular oxygen | F | 6 | GO:0005976 | polysaccharide metabolic process | P | 14 |
|  | GO:0016620 | oxidoreductase activity, acting on the aldehyde or oxo group of donors, NAD or NADP as acceptor | F | 9 | GO:0044262 | cellular carbohydrate metabolic process | P | 17 |
|  | GO:0016746 | transferase activity, transferring acyl groups | F | 23 |  |  |  |  |
|  | GO:1901606 | alpha-amino acid catabolic process | P | 11 |  |  |  |  |
|  | GO:0003333 | amino acid transmembrane transport | P | 12 |  |  |  |  |
|  | GO:0018874 | benzoate metabolic process | P | 4 |  |  |  |  |
|  | GO:0006631 | fatty acid metabolic process | P | 11 |  |  |  |  |
|  | GO:0006094 | gluconeogenesis | P | 11 |  |  |  |  |
|  | GO:0006096 | glycolytic process | P | 10 |  |  |  |  |
|  | GO:0006097 | glyoxylate cycle | P | 3 |  |  |  |  |
|  | GO:0005996 | monosaccharide metabolic process | P | 16 |  |  |  |  |
|  | GO:0046939 | nucleotide phosphorylation | P | 12 |  |  |  |  |
|  | GO:0055114 | oxidation-reduction process | P | 165 |  |  |  |  |
|  | GO:0042493 | response to drug | P | 4 |  |  |  |  |
|  | GO:0006099 | tricarboxylic acid cycle | P | 11 |  |  |  |  |
| l-6 | GO:0016021 | integral component of membrane | C | 90 | GO:0016021 | integral component of membrane | C | 75 |
|  | GO:0015171 | amino acid transmembrane transporter activity | F | 13 | GO:0031965 | nuclear membrane | C | 3 |
|  | GO:0005506 | iron ion binding | F | 35 | GO:0004553 | hydrolase activity, hydrolyzing O-glycosyl compounds | F | 33 |
|  | GO:0004497 | monooxygenase activity | F | 30 | GO:0003995 | acyl-CoA dehydrogenase activity | F | 7 |
|  | GO:0050660 | flavin adenine dinucleotide binding | F | 26 | GO:0016705 | oxidoreductase activity, acting on paired donors, with incorporation or reduction of molecular oxygen | F | 36 |
|  | GO:0016705 | oxidoreductase activity, acting on paired donors, with incorporation or reduction of molecular oxygen | F | 33 | GO:0005506 | iron ion binding | F | 34 |
|  | GO:0020037 | heme binding | F | 32 | GO:0004497 | monooxygenase activity | F | 29 |
|  | GO:0051213 | dioxygenase activity | F | 13 | GO:0051213 | dioxygenase activity | F | 13 |
|  | GO:0004553 | hydrolase activity, hydrolyzing O-glycosyl compounds | F | 26 | GO:0015171 | amino acid transmembrane transporter activity | F | 8 |
|  | GO:0016614 | oxidoreductase activity, acting on CH-OH group of donors | F | 21 | GO:0020037 | heme binding | F | 30 |
|  | GO:0016620 | oxidoreductase activity, acting on the aldehyde or oxo group of donors, NAD or NADP as acceptor | F | 7 | GO:0004003 | ATP-dependent DNA helicase activity | F | 5 |
|  | GO:0055114 | oxidation-reduction process | P | 142 | GO:0055114 | oxidation-reduction process | P | 120 |
|  | GO:0055085 | transmembrane transport | P | 78 | GO:0055085 | transmembrane transport | P | 68 |
|  | GO:0003333 | amino acid transmembrane transport | P | 13 | GO:0005975 | carbohydrate metabolic process | P | 49 |
|  | GO:0044282 | small molecule catabolic process | P | 18 | GO:0008643 | carbohydrate transport | P | 12 |
|  | GO:0042537 | benzene-containing compound metabolic process | P | 8 | GO:0003333 | amino acid transmembrane transport | P | 8 |
|  | GO:0032787 | monocarboxylic acid metabolic process | P | 24 | GO:0032508 | DNA duplex unwinding | P | 7 |
|  | GO:0071555 | cell wall organization | P | 5 |  |  |  |  |
|  | GO:0008643 | carbohydrate transport | P | 12 |  |  |  |  |
|  | GO:0006574 | valine catabolic process | P | 4 |  |  |  |  |
|  | GO:0006552 | leucine catabolic process | P | 4 |  |  |  |  |
|  | GO:0006550 | isoleucine catabolic process | P | 4 |  |  |  |  |
|  | GO:0005996 | monosaccharide metabolic process | P | 13 |  |  |  |  |
|  | GO:0042221 | response to chemical | P | 10 |  |  |  |  |
|  | GO:0015893 | drug transport | P | 3 |  |  |  |  |
|  | GO:0006546 | glycine catabolic process | P | 3 |  |  |  |  |
| l-c | GO:0016021 | integral component of membrane | C | 58 | GO:0051213 | dioxygenase activity | F | 11 |
|  | GO:0015171 | amino acid transmembrane transporter activity | F | 10 | GO:0016705 | oxidoreductase activity, acting on paired donors, with incorporation or reduction of molecular oxygen | F | 25 |
|  | GO:0051213 | dioxygenase activity | F | 12 | GO:0005506 | iron ion binding | F | 24 |
|  | GO:0050660 | flavin adenine dinucleotide binding | F | 19 | GO:0003995 | acyl-CoA dehydrogenase activity | F | 5 |
|  | GO:0016701 | oxidoreductase activity, acting on single donors with incorporation of molecular oxygen | F | 5 | GO:0004497 | monooxygenase activity | F | 20 |
|  | GO:0016620 | oxidoreductase activity, acting on the aldehyde or oxo group of donors, NAD or NADP as acceptor | F | 6 | GO:0022891 | substrate-specific transmembrane transporter activity | F | 23 |
|  | GO:0016614 | oxidoreductase activity, acting on CH-OH group of donors | F | 15 | GO:0004650 | polygalacturonase activity | F | 4 |
|  | GO:0016813 | hydrolase activity, acting on carbon-nitrogen (but not peptide) bonds, in linear amidines | F | 3 | GO:0015171 | amino acid transmembrane transporter activity | F | 6 |
|  | GO:0010181 | FMN binding | F | 7 | GO:0016701 | oxidoreductase activity, acting on single donors with incorporation of molecular oxygen | F | 5 |
|  | GO:0055114 | oxidation-reduction process | P | 95 | GO:0055114 | oxidation-reduction process | P | 82 |
|  | GO:0055085 | transmembrane transport | P | 50 | GO:0008643 | carbohydrate transport | P | 11 |
|  | GO:0003333 | amino acid transmembrane transport | P | 10 | GO:0055085 | transmembrane transport | P | 43 |
|  | GO:0044282 | small molecule catabolic process | P | 11 | GO:0044262 | cellular carbohydrate metabolic process | P | 14 |
|  | GO:1901606 | alpha-amino acid catabolic process | P | 9 | GO:0044282 | small molecule catabolic process | P | 10 |
|  | GO:0015893 | drug transport | P | 3 | GO:0005976 | polysaccharide metabolic process | P | 11 |
|  | GO:0018874 | benzoate metabolic process | P | 3 | GO:0003333 | amino acid transmembrane transport | P | 6 |
|  | GO:0042178 | xenobiotic catabolic process | P | 3 | GO:0071555 | cell wall organization | P | 4 |

Table s3. The enriched GO terms of down-regulated genes in liquid culture compare to 2, 4 and 6weeks after infection and the consistent down-regulated genes in bark. l-2(4,6,c)= liquid culture compare to 2-,4- and 6- weeks and CUGs, C= Cellular localization, P= Biological Process and F=Molecular Function

|  |  | *H. irregulare* |  |  |  | *H. occidentale* |  |  |
| --- | --- | --- | --- | --- | --- | --- | --- | --- |
|  | GO-ID | Term | Category | No. of genes | GO-ID | Term | Category | No. of genes |
| l-2 | GO:0009277 | fungal-type cell wall | C | 8 | GO:0050660 | flavin adenine dinucleotide binding | F | 34 |
|  | GO:0005199 | structural constituent of cell wall | F | 8 | GO:0016614 | oxidoreductase activity, acting on CH-OH group of donors | F | 27 |
|  | GO:0004185 | serine-type carboxypeptidase activity | F | 6 | GO:0020037 | heme binding | F | 32 |
|  | GO:0042546 | cell wall biogenesis | P | 8 | GO:0008762 | UDP-N-acetylmuramate dehydrogenase activity | F | 8 |
|  |  |  |  |  | GO:0016705 | oxidoreductase activity, acting on paired donors, with incorporation or reduction of molecular oxygen | F | 30 |
|  |  |  |  |  | GO:0055114 | oxidation-reduction process | P | 129 |
|  |  |  |  |  | GO:0006040 | amino sugar metabolic process | P | 13 |
| l-4 | GO:0009277 | fungal-type cell wall | C | 9 | GO:0050660 | flavin adenine dinucleotide binding | F | 28 |
|  | GO:0005576 | extracellular region | C | 9 | GO:0016614 | oxidoreductase activity, acting on CH-OH group of donors | F | 25 |
|  | GO:0004553 | hydrolase activity, hydrolyzing O-glycosyl compounds | F | 29 | GO:0020037 | heme binding | F | 31 |
|  | GO:0005199 | structural constituent of cell wall | F | 9 | GO:0004601 | peroxidase activity | F | 9 |
|  | GO:0008236 | serine-type peptidase activity | F | 11 | GO:0016705 | oxidoreductase activity, acting on paired donors, with incorporation or reduction of molecular oxygen | F | 28 |
|  | GO:0004185 | serine-type carboxypeptidase activity | F | 5 | GO:0008762 | UDP-N-acetylmuramate dehydrogenase activity | F | 8 |
|  | GO:0030247 | polysaccharide binding | F | 7 | GO:0004497 | monooxygenase activity | F | 23 |
|  | GO:0042546 | cell wall biogenesis | P | 9 | GO:0005506 | iron ion binding | F | 26 |
|  | GO:0005975 | carbohydrate metabolic process | P | 35 | GO:0055114 | oxidation-reduction process | P | 92 |
|  |  |  |  |  | GO:0006040 | amino sugar metabolic process | P | 10 |
|  |  |  |  |  | GO:0006979 | response to oxidative stress | P | 9 |
| l-6 | GO:0004185 | serine-type carboxypeptidase activity | F | 6 | GO:0050660 | flavin adenine dinucleotide binding | F | 28 |
|  | GO:0005199 | structural constituent of cell wall | F | 6 | GO:0016614 | oxidoreductase activity, acting on CH-OH group of donors | F | 25 |
|  | GO:0009277 | fungal-type cell wall | C | 6 | GO:0020037 | heme binding | F | 32 |
|  |  |  |  |  | GO:0004497 | monooxygenase activity | F | 25 |
|  |  |  |  |  | GO:0016705 | oxidoreductase activity, acting on paired donors, with incorporation or reduction of molecular oxygen | F | 29 |
|  |  |  |  |  | GO:0005506 | iron ion binding | F | 26 |
|  |  |  |  |  | GO:0008762 | UDP-N-acetylmuramate dehydrogenase activity | F | 7 |
|  |  |  |  |  | GO:0004671 | protein C-terminal S-isoprenylcysteine carboxyl O-methyltransferase activity | F | 5 |
|  |  |  |  |  | GO:0055114 | oxidation-reduction process | P | 104 |
|  |  |  |  |  | GO:0006040 | amino sugar metabolic process | P | 10 |
|  |  |  |  |  | GO:0006481 | C-terminal protein methylation | P | 5 |
| l-c | GO:0004185 | serine-type carboxypeptidase activity | F | 5 | GO:0050660 | flavin adenine dinucleotide binding | F | 21 |
|  | GO:0005199 | structural constituent of cell wall | F | 5 | GO:0016614 | oxidoreductase activity, acting on CH-OH group of donors | F | 19 |
|  | GO:0009277 | fungal-type cell wall | C | 5 | GO:0055114 | UDP-N-acetylmuramate dehydrogenase activity | F | 6 |
|  |  |  |  |  | GO:0008762 | oxidation-reduction process | P | 55 |

Table s4. List of common consistent up-regulated genes in bark of H. irregulare and H. occidentale. L=liquid culture 2w, 4w and 6w=2weeks, 4weeks and 6weeks (Unit is FPKM), L-2w, -4w and-6w =liquid culture compare with 2weeks, 4weeks and 6weeks (unit is log2 fold change)

|  | *H. irregulare* | | | | | | | *H. occidentale* | | | | | | |  |
| --- | --- | --- | --- | --- | --- | --- | --- | --- | --- | --- | --- | --- | --- | --- | --- |
| JGI-ID | L | 2w | 4w | 6w | L-2w | L-4w | L-6w | L | 2w | 4w | 6w | L-2w | L-4w | L-6w | Sequence description |
| 147564 | 69.64 | 4222.00 | 3546.45 | 2159.02 | 5.92 | 5.67 | 4.95 | 473.17 | 9887.28 | 4994.59 | 4810.55 | 4.39 | 3.40 | 3.35 | crispr-associated protein 1 |
| 442504 | 299.22 | 2385.03 | 980.85 | 1660.79 | 2.99 | 1.71 | 2.47 | 113.32 | 1235.95 | 455.09 | 722.00 | 3.45 | 2.01 | 2.67 | hypothetical protein |
| 408998 | 95.69 | 1689.00 | 1737.50 | 809.00 | 4.14 | 4.18 | 3.08 | 196.72 | 1075.61 | 624.87 | 618.73 | 2.45 | 1.67 | 1.65 | [Signal transduction mechanisms](http://genome.jgi.doe.gov/cgi-bin/kogBrowser?db=Hetan2&class=T&models=1) |
| 439015 | 109.28 | 1677.51 | 723.23 | 783.02 | 3.94 | 2.73 | 2.84 | 93.65 | 371.69 | 394.21 | 382.88 | 1.99 | 2.07 | 2.03 | hypothetical protein |
| 435092 | 5.73 | 1619.03 | 854.94 | 1390.33 | 8.14 | 7.22 | 7.92 | 28.52 | 682.55 | 671.84 | 1058.84 | 4.58 | 4.56 | 5.21 | alpha-ketoglutarate catabolism dioxygenase |
| 482007 | 124.96 | 1574.37 | 626.17 | 843.13 | 3.66 | 2.33 | 2.75 | 156.05 | 4433.07 | 2316.53 | 2502.23 | 4.83 | 3.89 | 4.00 | hypothetical protein |
| 62980 | 4.67 | 1406.37 | 253.68 | 593.14 | 8.24 | 5.76 | 6.99 | 19.60 | 682.60 | 117.97 | 115.92 | 5.12 | 2.59 | 2.56 | metalloprotease |
| 56025 | 12.68 | 1350.60 | 273.86 | 508.04 | 6.73 | 4.43 | 5.32 | 5.90 | 953.71 | 192.85 | 211.49 | 7.34 | 5.03 | 5.16 | peptidyl-lys metalloendopeptidase |
| 442642 | 142.33 | 1328.27 | 442.53 | 765.72 | 3.22 | 1.64 | 2.43 | 180.84 | 1131.04 | 517.18 | 480.95 | 2.64 | 1.52 | 1.41 | aldo keto reductase |
| 106809 | 70.72 | 1215.92 | 722.47 | 860.34 | 4.10 | 3.35 | 3.60 | 137.01 | 1153.79 | 1908.33 | 615.18 | 3.07 | 3.80 | 2.17 | general substrate transporter |
| 477946 | 227.24 | 1127.93 | 833.90 | 446.14 | 2.31 | 1.88 | 0.97 | 95.00 | 582.85 | 263.00 | 230.99 | 2.62 | 1.47 | 1.28 | ssp(prenyltransferase) |
| 119423 | 9.00 | 1020.15 | 210.05 | 1057.87 | 6.82 | 4.54 | 6.88 | 6.32 | 1665.65 | 1130.61 | 905.85 | 8.04 | 7.48 | 7.16 | multi-copper oxidase laccase-like protein |
| 452244 | 57.22 | 988.22 | 234.59 | 370.16 | 4.11 | 2.04 | 2.69 | 107.97 | 682.30 | 266.58 | 242.85 | 2.66 | 1.30 | 1.17 | nad-p-binding protein |
| 315329 | 52.77 | 853.68 | 771.22 | 784.52 | 4.02 | 3.87 | 3.89 | 39.34 | 530.09 | 540.24 | 627.91 | 3.75 | 3.78 | 4.00 | alpha-ketoglutarate-dependent sulfonate dioxygenase |
| 447745 | 25.57 | 790.64 | 278.09 | 551.53 | 4.95 | 3.44 | 4.43 | 2.90 | 1016.25 | 1038.94 | 1022.69 | 8.45 | 8.49 | 8.46 | hypothetical protein |
| 66124 | 33.57 | 742.80 | 2083.36 | 681.88 | 4.47 | 5.96 | 4.34 | 88.94 | 767.27 | 1330.93 | 1179.78 | 3.11 | 3.90 | 3.73 | abc transporter |
| 434890 | 42.96 | 722.43 | 487.35 | 445.19 | 4.07 | 3.50 | 3.37 | 90.81 | 272.66 | 323.32 | 180.34 | 1.59 | 1.83 | 0.99 | pali-domain-containing protein |
| 386881 | 83.72 | 713.44 | 450.51 | 419.34 | 3.09 | 2.43 | 2.32 | 134.48 | 600.81 | 508.78 | 517.66 | 2.16 | 1.92 | 1.94 | 2-nitropropane dioxygenase |
| 67601 | 7.75 | 688.22 | 84.78 | 351.33 | 6.47 | 3.45 | 5.50 | 8.37 | 422.51 | 84.52 | 129.29 | 5.66 | 3.34 | 3.95 | laccase |
| 126495 | 14.40 | 626.46 | 636.16 | 726.92 | 5.44 | 5.47 | 5.66 | 5.79 | 129.28 | 279.61 | 202.36 | 4.48 | 5.59 | 5.13 | agmatinase |
| 157466 | 58.47 | 592.21 | 194.50 | 376.69 | 3.34 | 1.73 | 2.69 | 25.80 | 473.83 | 130.73 | 120.54 | 4.20 | 2.34 | 2.22 | enolase c-terminal domain-like protein |
| 418837 | 7.36 | 591.85 | 321.49 | 372.35 | 6.33 | 5.45 | 5.66 | 3.77 | 442.03 | 207.27 | 194.32 | 6.87 | 5.78 | 5.69 | hypothetical protein |
| 384796 | 132.04 | 556.50 | 707.14 | 374.58 | 2.08 | 2.42 | 1.50 | 91.08 | 425.91 | 400.69 | 282.45 | 2.23 | 2.14 | 1.63 | flavocytochrome c |
| 460700 | 4.27 | 542.67 | 563.81 | 314.31 | 6.99 | 7.04 | 6.20 | 1.54 | 413.77 | 767.31 | 287.33 | 8.07 | 8.97 | 7.55 | integral membrane protein |
| 52748 | 6.76 | 529.03 | 514.61 | 323.48 | 6.29 | 6.25 | 5.58 | 12.46 | 136.99 | 235.37 | 268.61 | 3.46 | 4.24 | 4.43 | clavaminate synthase-like protein |
| NA | 14.59 | 496.80 | 139.94 | 967.23 | 5.09 | 3.26 | 6.05 | 3.75 | 1565.25 | 1667.34 | 1808.63 | 8.71 | 8.80 | 8.91 | barwin-like endoglucanase |
| 433781 | 56.74 | 463.00 | 181.57 | 215.83 | 3.03 | 1.68 | 1.93 | 129.00 | 485.28 | 384.36 | 323.65 | 1.91 | 1.58 | 1.33 | acyl- dehydrogenase nm domain-like protein |
| 42076 | 13.87 | 455.83 | 72.05 | 224.93 | 5.04 | 2.38 | 4.02 | 14.27 | 138.46 | 69.11 | 63.57 | 3.28 | 2.28 | 2.16 | glycoside hydrolase family 28 protein |
| 474812 | 113.10 | 453.08 | 352.78 | 461.28 | 2.00 | 1.64 | 2.03 | 12.89 | 202.79 | 125.23 | 146.81 | 3.98 | 3.28 | 3.51 | hypothetical protein HETIRDRAFT_474812 |
| 430720 | 9.92 | 420.96 | 1129.78 | 582.25 | 5.41 | 6.83 | 5.87 | 5.54 | 121.16 | 18.42 | 41.54 | 4.45 | 1.73 | 2.91 | hypothetical protein HETIRDRAFT_430720 |
| 457393 | 7.67 | 406.13 | 94.71 | 34.65 | 5.73 | 3.63 | 2.18 | 8.36 | 378.08 | 31.46 | 42.65 | 5.50 | 1.91 | 2.35 | hypothetical protein HETIRDRAFT_457393 |
| 470027 | 28.56 | 403.10 | 165.67 | 353.16 | 3.82 | 2.54 | 3.63 | 31.03 | 291.77 | 216.85 | 277.76 | 3.23 | 2.80 | 3.16 | thiolase-like protein |
| 106475 | 35.01 | 391.03 | 837.15 | 403.63 | 3.48 | 4.58 | 3.53 | 135.75 | 588.05 | 521.53 | 452.15 | 2.12 | 1.94 | 1.74 | Metallo-hydrolase/oxidoreductase |
| 148210 | 21.75 | 377.03 | 762.48 | 493.10 | 4.12 | 5.13 | 4.50 | 26.74 | 175.78 | 234.49 | 304.66 | 2.72 | 3.13 | 3.51 | cytochrome p450 |
| 37752 | 55.63 | 373.99 | 333.59 | 413.24 | 2.75 | 2.58 | 2.89 | 15.58 | 125.47 | 169.63 | 131.99 | 3.01 | 3.44 | 3.08 | hypothetical protein HETIRDRAFT_37752 |
| 156784 | 52.78 | 368.03 | 372.29 | 570.56 | 2.80 | 2.82 | 3.43 | 8.83 | 154.70 | 279.26 | 173.45 | 4.13 | 4.98 | 4.30 | apc amino acid permease |
| 45549 | 2.62 | 366.28 | 69.89 | 43.24 | 7.13 | 4.74 | 4.05 | 5.05 | 323.07 | 40.47 | 14.68 | 6.00 | 3.00 | 1.54 | aminopeptidase |
| 63800 | 18.83 | 358.81 | 47.17 | 162.06 | 4.25 | 1.32 | 3.11 | 10.92 | 508.24 | 81.19 | 84.10 | 5.54 | 2.89 | 2.95 | glycoside hydrolase family 16 protein |
| 419381 | 18.83 | 358.81 | 47.17 | 162.06 | 4.25 | 1.32 | 3.11 | 10.92 | 508.24 | 81.19 | 84.10 | 5.54 | 2.89 | 2.95 | tetrahydrobiopterin biosynthesis enzymes-like |
| 243376 | 72.17 | 350.39 | 896.76 | 340.26 | 2.28 | 3.64 | 2.24 | 200.57 | 402.54 | 469.74 | 555.30 | 1.01 | 1.23 | 1.47 | hypothetical protein HETIRDRAFT_243376 |
| 409415 | 82.46 | 334.58 | 382.43 | 366.47 | 2.02 | 2.21 | 2.15 | 106.45 | 201.76 | 249.90 | 250.09 | 0.92 | 1.23 | 1.23 | hypothetical protein HETIRDRAFT_409415 |
| 438756 | 16.85 | 333.39 | 45.06 | 53.33 | 4.31 | 1.42 | 1.66 | 3.95 | 67.87 | 41.32 | 18.15 | 4.10 | 3.39 | 2.20 | serine protease s28 |
| 469444 | 88.07 | 323.26 | 193.02 | 245.52 | 1.88 | 1.13 | 1.48 | 38.20 | 318.18 | 122.37 | 124.47 | 3.06 | 1.68 | 1.70 | d-galacturonic acid reductase |
| 381841 | 79.74 | 312.87 | 573.68 | 225.33 | 1.97 | 2.85 | 1.50 | 92.73 | 321.54 | 193.41 | 222.41 | 1.79 | 1.06 | 1.26 | acyl- dehydrogenase nm domain-like protein |
| 127284 | 6.60 | 310.21 | 44.91 | 245.00 | 5.56 | 2.77 | 5.21 | 8.03 | 139.58 | 82.33 | 71.51 | 4.12 | 3.36 | 3.15 | family s53 protease |
| 429979 | 41.98 | 303.52 | 255.49 | 223.80 | 2.85 | 2.61 | 2.41 | 22.33 | 161.92 | 229.02 | 276.22 | 2.86 | 3.36 | 3.63 | o-acetylhomoserine ami |
| 319723 | 50.38 | 301.97 | 561.72 | 317.30 | 2.58 | 3.48 | 2.65 | 73.43 | 201.56 | 412.81 | 277.75 | 1.46 | 2.49 | 1.92 | alcohol dehydrogenase iv |
| 441362 | 21.46 | 290.37 | 242.47 | 166.30 | 3.76 | 3.50 | 2.95 | 10.10 | 194.66 | 72.51 | 65.04 | 4.27 | 2.84 | 2.69 | glycoside hydrolase family 27 protein |
| 424001 | 10.43 | 278.33 | 76.60 | 206.40 | 4.74 | 2.88 | 4.31 | 6.77 | 233.20 | 111.25 | 128.31 | 5.11 | 4.04 | 4.24 | endonuclease exonuclease phosphatase family |
| 66540 | 53.41 | 270.46 | 393.32 | 394.54 | 2.34 | 2.88 | 2.88 | 26.95 | 128.48 | 219.62 | 186.74 | 2.25 | 3.03 | 2.79 | ammonium transporter |
| 154409 | 52.12 | 269.50 | 120.99 | 156.14 | 2.37 | 1.22 | 1.58 | 50.93 | 274.70 | 139.81 | 141.76 | 2.43 | 1.46 | 1.48 | acetyl- synthetase-like protein |
| 157457 | 36.67 | 266.35 | 140.46 | 110.50 | 2.86 | 1.94 | 1.59 | 66.74 | 133.56 | 229.53 | 153.63 | 1.00 | 1.78 | 1.20 | mfs sugar transporter(hexose transporter) |
| 68311 | 36.14 | 264.91 | 369.15 | 305.18 | 2.87 | 3.35 | 3.08 | 17.57 | 116.99 | 279.83 | 197.06 | 2.74 | 3.99 | 3.49 | cytochrome p450 |
| 127365 | 67.40 | 263.76 | 182.23 | 154.17 | 1.97 | 1.43 | 1.19 | 60.48 | 234.73 | 122.65 | 143.18 | 1.96 | 1.02 | 1.24 | nad-p-binding protein |
| 407079 | 78.47 | 250.93 | 197.85 | 244.31 | 1.68 | 1.33 | 1.64 | 28.32 | 125.36 | 88.33 | 97.22 | 2.15 | 1.64 | 1.78 | g2 mitotic-specific cyclin cyb1 |
| 62767 | 60.64 | 250.08 | 169.78 | 257.44 | 2.04 | 1.49 | 2.09 | 27.90 | 227.98 | 331.83 | 190.77 | 3.03 | 3.57 | 2.77 | mfs general substrate transporter |
| 68460 | 56.03 | 242.31 | 296.36 | 213.42 | 2.11 | 2.40 | 1.93 | 62.40 | 146.01 | 147.89 | 120.33 | 1.23 | 1.24 | 0.95 | adenine nucleotide transporter |
| 156655 | 14.08 | 239.34 | 80.35 | 187.80 | 4.09 | 2.51 | 3.74 | 19.09 | 152.30 | 203.32 | 154.57 | 3.00 | 3.41 | 3.02 | fad nad-p-binding domain-containing protein |
| 456869 | 17.84 | 239.15 | 75.73 | 149.10 | 3.74 | 2.09 | 3.06 | 15.13 | 341.93 | 120.85 | 122.75 | 4.50 | 3.00 | 3.02 | nad-p-binding protein |
| 315128 | 7.82 | 228.48 | 52.82 | 97.13 | 4.87 | 2.76 | 3.63 | 1.71 | 54.78 | 67.69 | 62.97 | 5.00 | 5.31 | 5.20 | serine protease |
| 480850 | 30.54 | 227.08 | 498.60 | 324.14 | 2.89 | 4.03 | 3.41 | 12.33 | 108.13 | 187.11 | 135.83 | 3.13 | 3.92 | 3.46 | protein |
| 325122 | 7.84 | 222.32 | 39.01 | 128.17 | 4.83 | 2.32 | 4.03 | 19.43 | 240.82 | 181.26 | 80.43 | 3.63 | 3.22 | 2.05 | aldo keto reductase |
| 49589 | 2.71 | 207.80 | 142.65 | 140.02 | 6.26 | 5.72 | 5.69 | 4.30 | 64.54 | 159.93 | 225.88 | 3.91 | 5.22 | 5.72 | mfs general substrate transporter |
| 164687 | 36.46 | 206.47 | 144.42 | 107.87 | 2.50 | 1.99 | 1.56 | 30.33 | 479.82 | 148.13 | 118.24 | 3.98 | 2.29 | 1.96 | glycoside hydrolase family 12 protein |
| 460686 | 15.14 | 203.54 | 49.53 | 76.30 | 3.75 | 1.71 | 2.33 | 5.44 | 83.25 | 43.24 | 46.41 | 3.94 | 2.99 | 3.09 | transmembrane protein |
| 458625 | 40.14 | 197.77 | 157.73 | 123.38 | 2.30 | 1.97 | 1.62 | 55.54 | 229.94 | 219.32 | 178.51 | 2.05 | 1.98 | 1.68 | multifunctional beta-oxidation protein |
| 482706 | 49.25 | 196.54 | 99.29 | 132.10 | 2.00 | 1.01 | 1.42 | 58.23 | 219.20 | 175.43 | 169.24 | 1.91 | 1.59 | 1.54 | 2-hydroxyacid dehydrogenase |
| 444923 | 51.54 | 192.46 | 100.24 | 176.65 | 1.90 | 0.96 | 1.78 | 20.84 | 138.41 | 65.16 | 57.52 | 2.73 | 1.64 | 1.46 | short-chain dehydrogenase |
| 439152 | 60.08 | 184.06 | 165.42 | 240.05 | 1.62 | 1.46 | 2.00 | 75.59 | 155.23 | 229.12 | 216.34 | 1.04 | 1.60 | 1.52 | nad-aldehyde dehydrogenase |
| 57251 | 4.69 | 180.48 | 78.37 | 238.80 | 5.27 | 4.06 | 5.67 | 8.62 | 446.73 | 201.58 | 50.44 | 5.70 | 4.55 | 2.55 | adenylation-thiolation-dehydrogenase |
| 157104 | 21.26 | 178.91 | 196.09 | 139.86 | 3.07 | 3.21 | 2.72 | 21.88 | 109.70 | 441.45 | 259.73 | 2.33 | 4.33 | 3.57 | homogentisate -dioxygenase |
| 448227 | 58.55 | 176.23 | 199.46 | 196.92 | 1.59 | 1.77 | 1.75 | 41.37 | 149.77 | 147.92 | 151.66 | 1.86 | 1.84 | 1.87 | fyve-domain-containing protein |
| 426430 | 70.75 | 167.82 | 423.07 | 180.30 | 1.25 | 2.58 | 1.35 | 70.57 | 226.57 | 220.43 | 275.56 | 1.68 | 1.64 | 1.97 | hypothetical protein HETIRDRAFT_426430 |
| 442731 | 43.93 | 165.75 | 97.00 | 101.57 | 1.92 | 1.14 | 1.21 | 34.27 | 118.03 | 83.73 | 100.06 | 1.78 | 1.29 | 1.55 | protein kinase subdomain-containing protein |
| 56922 | 45.13 | 162.90 | 132.36 | 208.63 | 1.85 | 1.55 | 2.21 | 27.90 | 727.53 | 551.87 | 908.64 | 4.70 | 4.31 | 5.03 | esterase lipase thioesterase |
| 314566 | 33.90 | 162.79 | 135.08 | 110.99 | 2.26 | 1.99 | 1.71 | 31.66 | 125.45 | 132.69 | 126.30 | 1.99 | 2.07 | 2.00 | mitochondrial acyl- dehydrogenase |
| 156259 | 33.27 | 162.40 | 106.03 | 117.39 | 2.29 | 1.67 | 1.82 | 46.99 | 128.37 | 118.09 | 112.72 | 1.45 | 1.33 | 1.26 | acyltransferase ctase cot cpt |
| 66031 | 43.16 | 161.59 | 185.63 | 250.36 | 1.90 | 2.10 | 2.54 | 30.04 | 97.89 | 195.27 | 129.63 | 1.70 | 2.70 | 2.11 | apc amino acid permease |
| 245172 | 54.02 | 160.07 | 117.11 | 174.18 | 1.57 | 1.12 | 1.69 | 40.30 | 130.75 | 105.34 | 110.82 | 1.70 | 1.39 | 1.46 | transcription factor activity |
| 435012 | 57.25 | 158.87 | 117.88 | 147.33 | 1.47 | 1.04 | 1.36 | 65.90 | 181.89 | 141.52 | 131.62 | 1.46 | 1.10 | 1.00 | hypothetical protein HETIRDRAFT_435012 |
| 173308 | 5.85 | 157.45 | 37.00 | 46.48 | 4.75 | 2.66 | 2.99 | 9.40 | 79.38 | 58.45 | 115.40 | 3.08 | 2.64 | 3.62 | acid protease |
| 148158 | 11.86 | 151.83 | 236.17 | 112.73 | 3.68 | 4.32 | 3.25 | 10.61 | 55.01 | 82.88 | 39.58 | 2.37 | 2.97 | 1.90 | carbohydrate-binding module family 13 protein |
| 437877 | 49.74 | 151.77 | 117.07 | 148.87 | 1.61 | 1.23 | 1.58 | 28.82 | 70.23 | 108.80 | 105.02 | 1.29 | 1.92 | 1.87 | succinate semialdehyde dehydrogenase |
| 157306 | 18.83 | 149.54 | 111.96 | 120.35 | 2.99 | 2.57 | 2.68 | 15.66 | 43.72 | 86.58 | 95.04 | 1.48 | 2.47 | 2.60 | amino acid transporter |
| 100517 | 4.73 | 146.90 | 15.90 | 72.08 | 4.96 | 1.75 | 3.93 | 1.59 | 46.18 | 21.01 | 43.50 | 4.86 | 3.72 | 4.77 | hypothetical protein STEHIDRAFT_154584 |
| 46814 | 2.43 | 143.99 | 136.07 | 84.17 | 5.89 | 5.81 | 5.12 | 3.73 | 32.58 | 66.61 | 90.80 | 3.13 | 4.16 | 4.60 | allantoate permease |
| 146089 | 50.13 | 143.06 | 413.68 | 133.32 | 1.51 | 3.04 | 1.41 | 84.48 | 171.44 | 323.33 | 300.28 | 1.02 | 1.94 | 1.83 | enth-domain-containing protein |
| 48830 | 6.22 | 142.54 | 53.94 | 161.19 | 4.52 | 3.12 | 4.69 | 3.36 | 96.75 | 36.68 | 37.62 | 4.85 | 3.45 | 3.49 | general substrate transporter |
| 317931 | 26.93 | 142.17 | 165.54 | 149.49 | 2.40 | 2.62 | 2.47 | 13.91 | 104.23 | 58.57 | 60.97 | 2.91 | 2.07 | 2.13 | dao-domain-containing protein |
| 108657 | 7.69 | 133.88 | 67.44 | 32.06 | 4.12 | 3.13 | 2.06 | 29.39 | 86.26 | 115.81 | 208.12 | 1.55 | 1.98 | 2.82 | fad nad-p-binding domain-containing protein |
| 306071 | 7.91 | 128.29 | 35.34 | 107.11 | 4.02 | 2.16 | 3.76 | 13.56 | 1245.55 | 497.40 | 519.84 | 6.52 | 5.20 | 5.26 | ketol-acid reductoisomerase activity |
| 313075 | 26.31 | 124.57 | 59.80 | 89.73 | 2.24 | 1.18 | 1.77 | 19.13 | 71.68 | 53.18 | 60.52 | 1.91 | 1.47 | 1.66 | acyl- dehydrogenase |
| 64456 | 14.41 | 123.57 | 58.61 | 82.52 | 3.10 | 2.02 | 2.52 | 9.98 | 189.40 | 163.72 | 191.80 | 4.25 | 4.04 | 4.26 | acid protease |
| 458479 | 36.76 | 122.43 | 102.01 | 188.82 | 1.74 | 1.47 | 2.36 | 9.89 | 46.01 | 76.82 | 95.27 | 2.22 | 2.96 | 3.27 | terpene synthase |
| 321457 | 29.90 | 117.63 | 204.67 | 212.72 | 1.98 | 2.78 | 2.83 | 32.27 | 163.97 | 71.52 | 81.56 | 2.35 | 1.15 | 1.34 | cytochrome p450 |
| 324961 | 19.42 | 114.12 | 100.86 | 101.09 | 2.55 | 2.38 | 2.38 | 17.32 | 55.00 | 50.95 | 39.80 | 1.67 | 1.56 | 1.20 | duf221-domain-containing protein |
| 315982 | 40.41 | 110.90 | 158.99 | 273.73 | 1.46 | 1.98 | 2.76 | 1.88 | 6.84 | 17.49 | 23.42 | 1.87 | 3.22 | 3.64 | acetamidase formamidase |
| 58302 | 14.18 | 110.86 | 50.20 | 56.13 | 2.97 | 1.82 | 1.98 | 3.84 | 13.62 | 25.60 | 41.85 | 1.83 | 2.74 | 3.45 | mfs general substrate transporter |
| 21356 | 18.08 | 109.72 | 78.95 | 78.59 | 2.60 | 2.13 | 2.12 | 31.05 | 116.77 | 94.57 | 94.18 | 1.91 | 1.61 | 1.60 | acetyl- synthetase-like protein |
| 460901 | 14.43 | 108.65 | 138.49 | 134.06 | 2.91 | 3.26 | 3.22 | 28.74 | 57.24 | 107.21 | 142.17 | 0.99 | 1.90 | 2.31 | peroxisomal copper amine oxidase |
| 407947 | 20.90 | 106.24 | 68.79 | 172.34 | 2.35 | 1.72 | 3.04 | 16.08 | 127.69 | 105.55 | 85.29 | 2.99 | 2.71 | 2.41 | hypothetical protein HETIRDRAFT_407947 |
| 11198 | 12.47 | 102.54 | 192.05 | 274.62 | 3.04 | 3.94 | 4.46 | 2.88 | 42.96 | 119.30 | 78.27 | 3.90 | 5.37 | 4.76 | apc amino acid permease |
| 67887 | 15.19 | 94.87 | 95.95 | 75.23 | 2.64 | 2.66 | 2.31 | 25.25 | 256.43 | 90.03 | 103.70 | 3.34 | 1.83 | 2.04 | hypothetical protein STEHIDRAFT_132894 |
| 447475 | 40.94 | 92.97 | 94.46 | 85.44 | 1.18 | 1.21 | 1.06 | 25.24 | 48.04 | 78.18 | 49.32 | 0.93 | 1.63 | 0.97 | hypothetical protein HETIRDRAFT_447475 |
| 244485 | 4.40 | 84.56 | 45.65 | 40.44 | 4.26 | 3.38 | 3.20 | 10.57 | 43.09 | 59.28 | 63.44 | 2.03 | 2.49 | 2.58 | transcription factor activity, |
| 331039 | 21.89 | 84.27 | 163.43 | 83.50 | 1.94 | 2.90 | 1.93 | 8.82 | 33.44 | 100.67 | 114.50 | 1.92 | 3.51 | 3.70 | clavaminate synthase-like protein |
| 156110 | 14.73 | 82.52 | 71.33 | 66.55 | 2.49 | 2.28 | 2.18 | 16.11 | 77.04 | 76.36 | 64.01 | 2.26 | 2.24 | 1.99 | carnitine acetyl transferase |
| 314072 | 15.22 | 82.51 | 160.90 | 105.22 | 2.44 | 3.40 | 2.79 | 11.64 | 58.57 | 38.51 | 52.18 | 2.33 | 1.73 | 2.16 | hypothetical protein HETIRDRAFT_314072 |
| 435834 | 14.17 | 77.21 | 57.42 | 146.29 | 2.45 | 2.02 | 3.37 | 1.16 | 42.73 | 52.89 | 33.46 | 5.21 | 5.52 | 4.86 | hypothetical protein HETIRDRAFT_435834 |
| 64447 | 21.95 | 72.76 | 59.26 | 78.71 | 1.73 | 1.43 | 1.84 | 7.12 | 40.70 | 60.11 | 41.21 | 2.52 | 3.08 | 2.53 | carboxypeptidase s |
| 163436 | 4.45 | 70.92 | 50.00 | 65.85 | 3.99 | 3.49 | 3.89 | 22.74 | 91.23 | 118.43 | 277.54 | 2.00 | 2.38 | 3.61 | amino acid transporter |
| 458734 | 19.23 | 70.20 | 53.60 | 88.39 | 1.87 | 1.48 | 2.20 | 8.56 | 38.56 | 37.69 | 32.89 | 2.17 | 2.14 | 1.94 | hypothetical protein HETIRDRAFT_458734 |
| 103954 | 27.34 | 68.15 | 58.51 | 69.56 | 1.32 | 1.10 | 1.35 | 23.66 | 53.90 | 68.76 | 51.87 | 1.19 | 1.54 | 1.13 | glycine cleavage system t protein |
| 58167 | 9.69 | 65.92 | 33.64 | 40.63 | 2.77 | 1.80 | 2.07 | 17.51 | 57.69 | 47.51 | 33.40 | 1.72 | 1.44 | 0.93 | glycoside hydrolase family 79 protein |
| 101142 | 9.76 | 63.61 | 54.65 | 57.23 | 2.70 | 2.49 | 2.55 | 4.71 | 15.65 | 19.39 | 20.47 | 1.73 | 2.04 | 2.12 | mfs general substrate transporter |
| 57042 | 19.04 | 63.54 | 102.99 | 68.47 | 1.74 | 2.44 | 1.85 | 23.77 | 86.31 | 130.12 | 111.48 | 1.86 | 2.45 | 2.23 | alpha beta-hydrolase |
| 51087 | 12.00 | 63.44 | 77.92 | 65.57 | 2.40 | 2.70 | 2.45 | 9.39 | 24.68 | 43.01 | 19.74 | 1.39 | 2.20 | 1.07 | von willebrand factor type a domain protein, |
| 103441 | 21.24 | 61.07 | 119.32 | 47.75 | 1.52 | 2.49 | 1.17 | 26.04 | 98.52 | 95.39 | 115.53 | 1.92 | 1.87 | 2.15 | hypothetical protein HETIRDRAFT_477615 |
| 426291 | 10.22 | 60.88 | 68.09 | 96.19 | 2.57 | 2.74 | 3.23 | 17.90 | 92.10 | 106.35 | 110.82 | 2.36 | 2.57 | 2.63 | transcriptional repressor xbp1 |
| 123163 | 16.23 | 60.83 | 144.28 | 181.64 | 1.91 | 3.15 | 3.48 | 5.08 | 40.31 | 32.73 | 61.96 | 2.99 | 2.69 | 3.61 | cytochrome p450 |
| 147890 | 23.89 | 60.37 | 66.44 | 65.15 | 1.34 | 1.48 | 1.45 | 16.34 | 47.32 | 32.47 | 36.80 | 1.53 | 0.99 | 1.17 | hypothetical protein HETIRDRAFT_147890 |
| 108165 | 5.45 | 60.02 | 19.14 | 43.40 | 3.46 | 1.81 | 2.99 | 9.77 | 86.63 | 34.90 | 38.14 | 3.15 | 1.84 | 1.96 | secreted protein |
| 450101 | 13.58 | 57.15 | 80.80 | 76.67 | 2.07 | 2.57 | 2.50 | 7.87 | 32.35 | 62.48 | 39.30 | 2.04 | 2.99 | 2.32 | mfs general substrate transporter |
| 474219 | 16.12 | 57.07 | 71.79 | 53.69 | 1.82 | 2.15 | 1.74 | 5.88 | 21.90 | 22.79 | 25.66 | 1.90 | 1.96 | 2.13 | hypothetical protein HETIRDRAFT_474219 |
| 447470 | 4.95 | 54.08 | 32.63 | 43.73 | 3.45 | 2.72 | 3.14 | 5.25 | 66.77 | 50.84 | 57.35 | 3.67 | 3.28 | 3.45 | hypothetical protein HETIRDRAFT_447470 |
| 167573 | 16.03 | 51.15 | 65.99 | 156.01 | 1.67 | 2.04 | 3.28 | 7.24 | 20.55 | 23.92 | 37.82 | 1.50 | 1.72 | 2.38 | terpenoid synthase |
| 321340 | 17.56 | 49.39 | 122.69 | 60.59 | 1.49 | 2.81 | 1.79 | 5.95 | 83.21 | 100.36 | 125.10 | 3.81 | 4.08 | 4.39 | hypothetical protein STEHIDRAFT_159587 |
| 447337 | 3.30 | 48.21 | 116.67 | 41.78 | 3.87 | 5.14 | 3.66 | 6.57 | 43.53 | 102.39 | 80.17 | 2.73 | 3.96 | 3.61 | hypothetical protein HETIRDRAFT_447337 |
| 476346 | 8.16 | 46.06 | 29.42 | 64.83 | 2.50 | 1.85 | 2.99 | 15.61 | 118.04 | 135.06 | 139.57 | 2.92 | 3.11 | 3.16 | fad nad-p-binding domain-containing protein |
| 101799 | 10.28 | 45.47 | 35.84 | 32.66 | 2.15 | 1.80 | 1.67 | 42.74 | 122.50 | 98.39 | 95.34 | 1.52 | 1.20 | 1.16 | hypothetical protein HETIRDRAFT_61766 |
| 481727 | 8.50 | 45.42 | 45.79 | 35.51 | 2.42 | 2.43 | 2.06 | 6.46 | 14.55 | 16.98 | 20.92 | 1.17 | 1.40 | 1.70 | acyltransferase ctase cot cpt |
| 439383 | 21.72 | 44.14 | 53.01 | 48.62 | 1.02 | 1.29 | 1.16 | 4.57 | 10.91 | 23.53 | 14.51 | 1.25 | 2.36 | 1.67 | fad nad-p-binding domain-containing protein |
| 247833 | 6.44 | 34.43 | 20.72 | 28.19 | 2.42 | 1.69 | 2.13 | 5.53 | 33.16 | 28.38 | 33.76 | 2.58 | 2.36 | 2.61 | hypothetical protein HETIRDRAFT_247833 |
| 156533 | 6.44 | 32.72 | 16.40 | 28.18 | 2.34 | 1.35 | 2.13 | 7.41 | 26.20 | 21.50 | 23.12 | 1.82 | 1.54 | 1.64 | protein |
| 50006 | 3.62 | 31.38 | 16.62 | 20.76 | 3.12 | 2.20 | 2.52 | 1.89 | 23.08 | 32.68 | 26.34 | 3.61 | 4.11 | 3.80 | fad nad-binding domain-containing protein |
| 443060 | 4.96 | 30.18 | 16.37 | 22.39 | 2.61 | 1.72 | 2.18 | 0.85 | 4.88 | 3.38 | 4.00 | 2.52 | 1.99 | 2.24 | transcription factor |
| 68483 | 14.06 | 28.17 | 53.85 | 27.65 | 1.00 | 1.94 | 0.98 | 14.27 | 37.79 | 43.52 | 32.45 | 1.40 | 1.61 | 1.19 | calcium-translocating p-type atpase |
| 470225 | 7.90 | 25.68 | 33.59 | 24.60 | 1.70 | 2.09 | 1.64 | 3.47 | 10.30 | 7.82 | 8.75 | 1.57 | 1.17 | 1.34 | protein serine threonine phosphatase 2c |
| 479585 | 4.22 | 24.70 | 24.33 | 27.16 | 2.55 | 2.53 | 2.68 | 6.48 | 15.85 | 17.99 | 16.51 | 1.29 | 1.47 | 1.35 | pkinase-domain-containing protein |
| 154778 | 8.13 | 22.16 | 28.42 | 37.95 | 1.45 | 1.81 | 2.22 | 7.27 | 19.34 | 29.76 | 25.77 | 1.41 | 2.03 | 1.83 | na(+) h(+) antiporter |
| 53419 | 7.14 | 17.93 | 19.41 | 26.09 | 1.33 | 1.44 | 1.87 | 14.07 | 33.25 | 27.52 | 28.10 | 1.24 | 0.97 | 1.00 | duf6-domain-containing protein |
| 170572 | 2.27 | 16.60 | 13.16 | 28.26 | 2.87 | 2.54 | 3.64 | 7.73 | 71.14 | 53.13 | 54.52 | 3.20 | 2.78 | 2.82 | carboxylesterase |
| 154865 | 3.31 | 13.67 | 24.51 | 15.93 | 2.04 | 2.89 | 2.26 | 0.26 | 8.33 | 9.96 | 15.63 | 4.97 | 5.23 | 5.88 | alpha beta-hydrolase |

Table s5. List of commonly consistent down regulated genes of H. irregulare and H. occidentale. L=liquid culture 2w, 4w and 6w=2weeks, 4weeks and 6weeks (Unit is FPKM), L-2w, -4w and-6w =liquid culture compare with 2weeks, 4weeks and 6weeks (unit is log2 fold change)

|  | *H. irregulare* | | | | | | | *H. occidentale* | | | | | | |  |
| --- | --- | --- | --- | --- | --- | --- | --- | --- | --- | --- | --- | --- | --- | --- | --- |
| JGI-ID | L | 2w | 4w | 6w | L-2w | L-4w | L-6w | L | 2w | 4w | 6w | L-2w | L-4w | L-6w | SeqDesc |
| 456720 | 602.60 | 124.90 | 57.18 | 68.66 | -2.27 | -3.40 | -3.13 | 472.16 | 69.71 | 51.44 | 48.45 | -2.76 | -3.20 | -3.28 | alpha beta-hydrolase |
| 306548 | 269.13 | 19.10 | 28.16 | 25.73 | -3.82 | -3.26 | -3.39 | 115.58 | 59.57 | 18.84 | 29.54 | -0.96 | -2.62 | -1.97 | nucleotide-sugar transporter |
| 41587 | 70.93 | 11.68 | 21.17 | 12.98 | -2.60 | -1.74 | -2.45 | 22.14 | 9.57 | 10.89 | 11.86 | -1.21 | -1.02 | -0.90 | polysaccharide lyase family 8 protein |
| 456967 | 1940.73 | 49.99 | 109.03 | 79.37 | -5.28 | -4.15 | -4.61 | 221.74 | 71.20 | 105.13 | 119.34 | -1.64 | -1.08 | -0.89 | 60s ribosome subunit biogenesis nip7 |
| 59406 | 334.27 | 25.18 | 34.98 | 41.00 | -3.73 | -3.26 | -3.03 | 136.83 | 52.54 | 59.18 | 53.32 | -1.38 | -1.21 | -1.36 | cytochrome p450 monooxygenase 52 |
| 305913 | 9478.38 | 747.33 | 732.63 | 439.81 | -3.66 | -3.69 | -4.43 | 5169.99 | 167.33 | 146.60 | 186.67 | -4.95 | -5.14 | -4.79 | copper transporter |
| 146116 | 567.27 | 10.12 | 7.80 | 8.10 | -5.81 | -6.18 | -6.13 | 13.98 | 1.36 | 2.74 | 2.48 | -3.37 | -2.35 | -2.49 | alpha beta-hydrolase |
| 60120 | 534.16 | 32.10 | 35.34 | 45.76 | -4.06 | -3.92 | -3.55 | 726.84 | 45.40 | 27.87 | 42.82 | -4.00 | -4.70 | -4.09 | alpha beta-hydrolase |
| 471672 | 401.55 | 31.63 | 36.80 | 34.51 | -3.67 | -3.45 | -3.54 | 115.70 | 59.09 | 49.74 | 61.51 | -0.97 | -1.22 | -0.91 | protein |
| 449337 | 56.31 | 10.12 | 7.36 | 9.27 | -2.48 | -2.93 | -2.60 | 9.82 | 3.50 | 3.02 | 3.80 | -1.49 | -1.70 | -1.37 | proline-rich protein |
| 62555 | 181.56 | 13.21 | 17.24 | 15.65 | -3.78 | -3.40 | -3.54 | 19.50 | 1.53 | 2.16 | 2.13 | -3.67 | -3.18 | -3.20 | deoxyribodipyrimidine photo-lyase |
| 171279 | 82.73 | 34.04 | 25.77 | 36.45 | -1.28 | -1.68 | -1.18 | 65.63 | 35.17 | 24.82 | 33.30 | -0.90 | -1.40 | -0.98 | sno glutamine amidotransferase |
| 473515 | 158.99 | 16.20 | 35.76 | 20.28 | -3.29 | -2.15 | -2.97 | 44.29 | 8.78 | 6.79 | 8.52 | -2.33 | -2.71 | -2.38 | nad-p-binding protein |
| 315451 | 360.95 | 113.25 | 152.40 | 111.63 | -1.67 | -1.24 | -1.69 | 263.96 | 77.99 | 84.93 | 80.62 | -1.76 | -1.64 | -1.71 | hypothetical protein GLOTRDRAFT_138559 |
| 316455 | 120.08 | 29.53 | 35.96 | 31.15 | -2.02 | -1.74 | -1.95 | 80.40 | 21.89 | 9.41 | 15.44 | -1.88 | -3.10 | -2.38 | hypothetical protein HETIRDRAFT_316455 |
| 315349 | 279.39 | 44.33 | 60.11 | 60.43 | -2.66 | -2.22 | -2.21 | 325.59 | 57.77 | 52.52 | 70.53 | -2.49 | -2.63 | -2.21 | duf427-domain-containing protein |
| 444640 | 90.80 | 5.41 | 13.38 | 16.94 | -4.07 | -2.76 | -2.42 | 114.95 | 7.52 | 11.56 | 12.87 | -3.93 | -3.31 | -3.16 | hypothetical protein HETIRDRAFT_444640 |
| 458718 | 58.00 | 9.36 | 23.68 | 24.35 | -2.63 | -1.29 | -1.25 | 32.70 | 13.48 | 9.52 | 12.93 | -1.28 | -1.78 | -1.34 | c6 transcription |
| 173067 | 88.43 | 7.12 | 9.33 | 8.98 | -3.63 | -3.24 | -3.30 | 51.10 | 12.52 | 6.02 | 7.77 | -2.03 | -3.09 | -2.72 | alpha beta-hydrolase |
| 474849 | 103.04 | 4.93 | 5.38 | 6.36 | -4.38 | -4.26 | -4.02 | 9.35 | 2.01 | 1.78 | 2.47 | -2.22 | -2.39 | -1.92 | fad-binding domain-containing protein |
| 36528 | 846.74 | 214.21 | 192.70 | 278.39 | -1.98 | -2.14 | -1.60 | 1008.25 | 62.11 | 154.68 | 102.57 | -4.02 | -2.70 | -3.30 | acid protease |
| 318087 | 33.06 | 7.25 | 11.59 | 1.43 | -2.19 | -1.51 | -4.53 | 7.31 | 2.88 | 2.13 | 2.59 | -1.35 | -1.78 | -1.50 | hypothetical protein HETIRDRAFT_318087 |
| 440061 | 361.52 | 153.72 | 124.77 | 146.35 | -1.23 | -1.53 | -1.30 | 295.71 | 136.00 | 100.41 | 120.56 | -1.12 | -1.56 | -1.29 | hypothetical protein HETIRDRAFT_440061 |
| 418027 | 40.69 | 15.34 | 15.41 | 18.75 | -1.41 | -1.40 | -1.12 | 52.16 | 15.14 | 18.05 | 12.23 | -1.78 | -1.53 | -2.09 | hypothetical protein HETIRDRAFT_418027 |
| 445021 | 121.77 | 17.87 | 18.09 | 28.63 | -2.77 | -2.75 | -2.09 | 566.05 | 31.47 | 22.98 | 19.23 | -4.17 | -4.62 | -4.88 | hypothetical protein HETIRDRAFT_445021 |
| 146178 | 913.07 | 77.36 | 132.51 | 253.18 | -3.56 | -2.78 | -1.85 | 62.54 | 24.86 | 16.03 | 7.69 | -1.33 | -1.96 | -3.02 | glycoside hydrolase family 16 protein |
| 181069 | 235.68 | 2.93 | 12.85 | 9.67 | -6.33 | -4.20 | -4.61 | 135.66 | 9.86 | 5.17 | 6.81 | -3.78 | -4.71 | -4.32 | manganese peroxidase |
| 146274 | 460.68 | 17.78 | 14.30 | 19.55 | -4.70 | -5.01 | -4.56 | 92.45 | 5.02 | 2.87 | 2.33 | -4.20 | -5.01 | -5.31 | subtilisin-like protein |
| 476506 | 864.32 | 336.14 | 344.63 | 357.55 | -1.36 | -1.33 | -1.27 | 2559.01 | 451.43 | 487.59 | 424.62 | -2.50 | -2.39 | -2.59 | hypothetical protein HETIRDRAFT_476506 |
| 320049 | 72.63 | 4.46 | 11.12 | 8.79 | -4.02 | -2.71 | -3.05 | 43.28 | 5.43 | 5.93 | 6.06 | -2.99 | -2.87 | -2.84 | protein serine threonine phosphatase 2c |
| 385801 | 87.07 | 40.89 | 37.52 | 41.20 | -1.09 | -1.21 | -1.08 | 103.45 | 44.44 | 38.41 | 32.76 | -1.22 | -1.43 | -1.66 | ferrochelatase |
| 419047 | 55.63 | 5.19 | 12.82 | 9.56 | -3.42 | -2.12 | -2.54 | 39.98 | 10.05 | 8.91 | 7.50 | -1.99 | -2.17 | -2.41 | hypothetical protein HETIRDRAFT_316690 |
| 452866 | 154.12 | 19.95 | 21.81 | 27.98 | -2.95 | -2.82 | -2.46 | 57.58 | 9.78 | 14.02 | 24.43 | -2.56 | -2.04 | -1.24 | acyl- n-acyltransferase |
| 445608 | 27.32 | 6.94 | 4.70 | 5.43 | -1.98 | -2.54 | -2.33 | 15.76 | 4.90 | 3.49 | 4.50 | -1.69 | -2.18 | -1.81 | protein prib |
| 64133 | 180.36 | 14.67 | 11.50 | 12.35 | -3.62 | -3.97 | -3.87 | 37.42 | 11.36 | 12.64 | 13.72 | -1.72 | -1.57 | -1.45 | zip-like iron-zinc transporter |
| 35909 | 725.36 | 217.21 | 128.67 | 217.39 | -1.74 | -2.50 | -1.74 | 593.18 | 51.85 | 92.82 | 79.58 | -3.52 | -2.68 | -2.90 | glutamate decarboxylase |
| 410823 | 85.34 | 3.96 | 6.35 | 11.12 | -4.43 | -3.75 | -2.94 | 11.32 | 1.42 | 1.24 | 1.93 | -3.00 | -3.19 | -2.55 | hypothetical protein HETIRDRAFT_410823 |
| 172810 | 232.05 | 18.36 | 3.78 | 55.91 | -3.66 | -5.94 | -2.05 | 299.69 | 68.93 | 70.71 | 56.98 | -2.12 | -2.08 | -2.39 | acid protease |
| 435869 | 608.30 | 49.87 | 108.04 | 110.89 | -3.61 | -2.49 | -2.46 | 622.19 | 67.90 | 36.84 | 74.40 | -3.20 | -4.08 | -3.06 | macrofage activating glycoprotein |
| 39061 | 544.92 | 16.44 | 14.64 | 25.32 | -5.05 | -5.22 | -4.43 | 38.66 | 3.90 | 2.59 | 4.34 | -3.31 | -3.90 | -3.15 | glycoside hydrolase family 47 protein |
| 163995 | 52.45 | 5.90 | 4.07 | 17.35 | -3.15 | -3.69 | -1.60 | 87.69 | 1.04 | 1.56 | 2.34 | -6.40 | -5.81 | -5.23 | hypothetical protein HETIRDRAFT_163995 |
| 68367 | 77.05 | 8.39 | 11.28 | 10.21 | -3.20 | -2.77 | -2.92 | 57.72 | 18.26 | 14.30 | 15.77 | -1.66 | -2.01 | -1.87 | serine carboxypeptidase |
| 143484 | 56.73 | 8.61 | 15.45 | 24.14 | -2.72 | -1.88 | -1.23 | 42.32 | 5.76 | 10.05 | 7.14 | -2.88 | -2.07 | -2.57 | nad-p-binding protein |
| 327286 | 58.17 | 7.73 | 12.00 | 19.08 | -2.91 | -2.28 | -1.61 | 115.15 | 4.57 | 3.67 | 2.59 | -4.66 | -4.97 | -5.47 | phosphoglycerate mutase-like protein |
| 421727 | 109.76 | 25.12 | 35.44 | 29.67 | -2.13 | -1.63 | -1.89 | 346.73 | 55.19 | 21.58 | 40.03 | -2.65 | -4.01 | -3.11 | fad-binding domain-containing protein |
| 460782 | 215.11 | 11.39 | 9.83 | 9.66 | -4.24 | -4.45 | -4.48 | 46.11 | 10.50 | 5.04 | 5.84 | -2.13 | -3.19 | -2.98 | hypothetical protein HETIRDRAFT_460782 |
| 105914 | 13.77 | 0.00 | 0.00 | 0.00 | #NAME | #NAME | #NAME | 140.41 | 1.70 | 2.59 | 1.21 | -6.37 | -5.76 | -6.86 | fungal hydrophobin |
| 442002 | 1077.66 | 2.98 | 40.51 | 10.20 | -8.50 | -4.73 | -6.72 | 321.27 | 10.40 | 19.97 | 8.46 | -4.95 | -4.01 | -5.25 | immunomodulatory protein |
| 328946 | 339.38 | 32.60 | 36.32 | 16.05 | -3.38 | -3.22 | -4.40 | 110.85 | 2.15 | 1.26 | 1.69 | -5.69 | -6.45 | -6.03 | immunomodulatory protein |
| 156743 | 197.97 | 8.21 | 9.25 | 8.35 | -4.59 | -4.42 | -4.57 | 68.35 | 4.51 | 5.34 | 4.39 | -3.92 | -3.68 | -3.96 | copper radical oxidase |
| 446505 | 466.18 | 8.62 | 70.13 | 52.12 | -5.76 | -2.73 | -3.16 | 379.63 | 18.16 | 12.13 | 9.39 | -4.39 | -4.97 | -5.34 | protein |
| 148119 | 18978.00 | 326.93 | 258.76 | 693.77 | -5.86 | -6.20 | -4.77 | 2013.10 | 154.01 | 13.83 | 21.03 | -3.71 | -7.19 | -6.58 | fungal hydrophobin |
| 181098 | 10000.30 | 143.49 | 246.26 | 513.67 | -6.12 | -5.34 | -4.28 | 2307.54 | 135.18 | 58.28 | 26.81 | -4.09 | -5.31 | -6.43 | fungal hydrophobin |
| 460967 | 74.59 | 19.07 | 16.48 | 22.91 | -1.97 | -2.18 | -1.70 | 42.99 | 19.52 | 17.63 | 20.54 | -1.14 | -1.29 | -1.07 | fungal zn -cys binuclear cluster domain |
| 152387 | 370.80 | 90.71 | 102.25 | 84.15 | -2.03 | -1.86 | -2.14 | 99.83 | 32.08 | 26.12 | 25.24 | -1.64 | -1.93 | -1.98 | gmc oxidoreductase |
| 148298 | 85.23 | 34.00 | 26.41 | 36.12 | -1.33 | -1.69 | -1.24 | 39.81 | 15.96 | 19.64 | 21.97 | -1.32 | -1.02 | -0.86 | glycoside hydrolase family 16 protein |
| 481612 | 636.37 | 229.19 | 180.02 | 190.85 | -1.47 | -1.82 | -1.74 | 130.65 | 50.69 | 21.00 | 28.16 | -1.37 | -2.64 | -2.21 | hypothetical protein HETIRDRAFT_481612 |
| 329914 | 147.48 | 53.96 | 48.87 | 58.66 | -1.45 | -1.59 | -1.33 | 166.91 | 36.52 | 48.14 | 55.97 | -2.19 | -1.79 | -1.58 | hypothetical protein HETIRDRAFT_329914 |
| 412440 | 164.79 | 52.84 | 84.85 | 72.46 | -1.64 | -0.96 | -1.19 | 164.44 | 62.20 | 79.07 | 76.18 | -1.40 | -1.06 | -1.11 | hypothetical protein HETIRDRAFT_412440 |
| 442473 | 18.20 | 5.50 | 3.30 | 3.26 | -1.73 | -2.46 | -2.48 | 8.48 | 1.70 | 1.49 | 2.72 | -2.32 | -2.50 | -1.64 | hypothetical protein HETIRDRAFT_442473 |
| 148913 | 519.77 | 141.43 | 122.54 | 127.97 | -1.88 | -2.08 | -2.02 | 407.08 | 111.91 | 105.15 | 176.20 | -1.86 | -1.95 | -1.21 | gmc oxidoreductase |
| 442533 | 1539.95 | 298.51 | 565.26 | 243.14 | -2.37 | -1.45 | -2.66 | 3126.58 | 102.25 | 703.82 | 293.08 | -4.93 | -2.15 | -3.42 |  |
| 482075 | 18.88 | 4.78 | 4.16 | 3.12 | -1.98 | -2.18 | -2.60 | 50.40 | 7.95 | 6.30 | 7.77 | -2.66 | -3.00 | -2.70 | d-amino-acid oxidase |
| 120243 | 32.41 | 2.76 | 3.52 | 4.16 | -3.56 | -3.20 | -2.96 | 59.69 | 6.11 | 5.49 | 3.53 | -3.29 | -3.44 | -4.08 | alcohol oxidase |
| 456021 | 82.13 | 26.31 | 26.56 | 26.48 | -1.64 | -1.63 | -1.63 | 79.49 | 13.69 | 20.56 | 12.42 | -2.54 | -1.95 | -2.68 | transmembrane protein |
| 330694 | 141.83 | 24.57 | 36.04 | 17.06 | -2.53 | -1.98 | -3.06 | 27.95 | 7.38 | 5.95 | 6.56 | -1.92 | -2.23 | -2.09 | mfs multidrug |
| 442605 | 458.47 | 23.62 | 7.72 | 7.54 | -4.28 | -5.89 | -5.93 | 1881.73 | 12.85 | 7.88 | 7.64 | -7.19 | -7.90 | -7.94 | hypothetical protein HETIRDRAFT_442605 |
| 442611 | 57.04 | 20.66 | 22.06 | 24.87 | -1.47 | -1.37 | -1.20 | 46.24 | 13.02 | 9.87 | 15.60 | -1.83 | -2.23 | -1.57 | hypothetical protein HETIRDRAFT_442611 |
| 149096 | 96.74 | 20.00 | 17.81 | 23.79 | -2.27 | -2.44 | -2.02 | 105.40 | 2.12 | 1.54 | 1.87 | -5.63 | -6.09 | -5.82 | proline-specific peptidase |
| 442717 | 128.81 | 50.92 | 59.10 | 64.56 | -1.34 | -1.12 | -1.00 | 89.96 | 33.99 | 43.50 | 37.57 | -1.40 | -1.05 | -1.26 | hypothetical protein HETIRDRAFT_164979 |

Table s6. List of CUGs specific to H. irregulare. L=liquid culture 2w, 4w and 6w=2weeks, 4weeks and 6weeks (Unit is FPKM), L-2w, -4w and-6w =liquid culture compare with 2weeks, 4weeks and 6weeks (unit is log2 fold change)

| JGI-ID | L | 2w | 4w | 6w | L-2w | L-4w | L-6w | SeqDesc |
| --- | --- | --- | --- | --- | --- | --- | --- | --- |
| 482290 | 8.06 | 1365.77 | 644.44 | 123.52 | 7.40 | 6.32 | 3.94 | small secreted protein |
| 436715 | 19.02 | 2165.06 | 221.92 | 549.84 | 6.83 | 3.54 | 4.85 | metallo peptidase m36 |
| 453968 | 2.82 | 244.87 | 385.79 | 27.83 | 6.44 | 7.10 | 3.31 | sam-dependent methyltransferase |
| 181240 | 14.38 | 1119.14 | 53.30 | 1008.72 | 6.28 | 1.89 | 6.13 | alcohol oxidase |
| 122112 | 8.86 | 562.85 | 225.05 | 231.68 | 5.99 | 4.67 | 4.71 | general substrate transporter |
| 172039 | 1.77 | 109.70 | 157.64 | 22.61 | 5.96 | 6.48 | 3.68 | laccase |
| 423383 | 74.69 | 3513.51 | 2999.22 | 4378.95 | 5.56 | 5.33 | 5.87 | transferase activity, transferring glycosyl groups |
| 126494 | 3.73 | 162.00 | 134.82 | 184.41 | 5.44 | 5.18 | 5.63 | mop flippase |
| 102327 | 3.97 | 165.77 | 188.17 | 298.91 | 5.39 | 5.57 | 6.24 | alcohol oxidase-like protein |
| 67956 | 6.76 | 231.11 | 32.97 | 85.11 | 5.09 | 2.29 | 3.65 | glycoside hydrolase family 43 protein |
| 482427 | 11.61 | 396.17 | 55.44 | 38.83 | 5.09 | 2.26 | 1.74 | probable serine threonine-protein kinase kinx- partial |
| 306610 | 7.07 | 229.61 | 187.23 | 250.26 | 5.02 | 4.73 | 5.15 | hypothetical protein HETIRDRAFT_306610 |
| 50287 | 23.35 | 665.31 | 98.38 | 225.43 | 4.83 | 2.07 | 3.27 | alpha beta-hydrolase |
| 149028 | 30.12 | 830.12 | 1582.71 | 646.77 | 4.78 | 5.72 | 4.42 | cytochrome p450 |
| 65122 | 15.52 | 413.42 | 486.28 | 165.77 | 4.73 | 4.97 | 3.42 | isocitrate lyase |
| 61667 | 16.50 | 427.32 | 276.23 | 439.51 | 4.69 | 4.07 | 4.74 | opt oligopeptide transporter |
| 445713 | 112.60 | 2676.25 | 731.36 | 533.86 | 4.57 | 2.70 | 2.25 | hypothetical protein HETIRDRAFT_445713 |
| 308436 | 10.19 | 237.49 | 190.08 | 158.46 | 4.54 | 4.22 | 3.96 | protein |
| 37102 | 24.73 | 504.44 | 1002.62 | 266.77 | 4.35 | 5.34 | 3.43 | transpoter? |
| 53448 | 15.58 | 313.32 | 1172.03 | 261.77 | 4.33 | 6.23 | 4.07 | general substrate transporter |
| 319827 | 11.88 | 236.96 | 51.59 | 149.83 | 4.32 | 2.12 | 3.66 | nuclease le1 |
| 410050 | 24.42 | 468.54 | 383.11 | 300.03 | 4.26 | 3.97 | 3.62 | had-like protein |
| 307854 | 11.20 | 184.96 | 178.11 | 314.26 | 4.05 | 3.99 | 4.81 | arginase deacetylase |
| 106177 | 41.07 | 674.61 | 2612.78 | 439.06 | 4.04 | 5.99 | 3.42 | ---NA--- |
| 152572 | 6.99 | 112.18 | 61.69 | 84.85 | 4.00 | 3.14 | 3.60 | aryl-alcohol dehydrogenase |
| 442505 | 42.60 | 627.20 | 1203.91 | 586.26 | 3.88 | 4.82 | 3.78 | nadh:flavin oxidoreductase nadh oxidase |
| 442648 | 39.62 | 583.30 | 208.19 | 514.29 | 3.88 | 2.39 | 3.70 | fibroin heavy chain-like |
| 428080 | 2.14 | 27.92 | 42.25 | 56.45 | 3.70 | 4.30 | 4.72 | hypothetical protein HETIRDRAFT_428080 |
| 427131 | 17.98 | 229.98 | 233.07 | 286.40 | 3.68 | 3.70 | 3.99 | hypothetical protein HETIRDRAFT_427131 |
| 101859 | 10.06 | 125.68 | 108.83 | 95.45 | 3.64 | 3.44 | 3.25 | hypothetical protein HETIRDRAFT_101859 |
| 124446 | 28.19 | 350.43 | 73.51 | 139.01 | 3.64 | 1.38 | 2.30 | aldehyde dehydrogenase |
| 39375 | 17.38 | 208.60 | 114.28 | 146.32 | 3.59 | 2.72 | 3.07 | ferric reductase |
| 448476 | 20.87 | 242.30 | 112.05 | 193.50 | 3.54 | 2.42 | 3.21 | rhamnose mutarotase |
| 126669 | 8.70 | 97.00 | 153.89 | 211.68 | 3.48 | 4.14 | 4.60 | ncs1 nucleoside transporter family |
| 473974 | 5.91 | 64.54 | 31.13 | 32.14 | 3.45 | 2.40 | 2.44 | nad-p-binding protein |
| 427791 | 46.40 | 471.00 | 336.17 | 264.76 | 3.34 | 2.86 | 2.51 | mismatched base pair and cruciform dna recognition protein |
| 68439 | 36.71 | 363.00 | 124.28 | 107.90 | 3.31 | 1.76 | 1.56 | alcohol oxidase |
| 414947 | 50.31 | 494.29 | 790.03 | 659.89 | 3.30 | 3.97 | 3.71 | hypothetical protein HETIRDRAFT_414947 |
| 157257 | 61.21 | 576.44 | 763.55 | 177.37 | 3.24 | 3.64 | 1.53 | alpha beta-hydrolase |
| 451269 | 5.94 | 55.45 | 55.70 | 44.95 | 3.22 | 3.23 | 2.92 | hypothetical protein HETIRDRAFT_451269 |
| 68105 | 14.64 | 135.36 | 249.20 | 59.13 | 3.21 | 4.09 | 2.01 | mitochondrial carrier |
| 163506 | 44.65 | 407.47 | 808.16 | 508.37 | 3.19 | 4.18 | 3.51 | hypothetical protein HETIRDRAFT_163506 |
| 59424 | 21.14 | 185.55 | 173.56 | 429.48 | 3.13 | 3.04 | 4.34 | mfs general substrate transporter |
| 67820 | 48.26 | 405.63 | 209.12 | 377.42 | 3.07 | 2.12 | 2.97 | alcohol oxidase |
| 384154 | 399.98 | 3230.78 | 2242.54 | 1639.10 | 3.01 | 2.49 | 2.03 | hypothetical protein HETIRDRAFT_384154, partial |
| 452516 | 46.77 | 370.58 | 427.44 | 197.24 | 2.99 | 3.19 | 2.08 | hypothetical protein HETIRDRAFT_452516 |
| 385524 | 78.70 | 594.17 | 624.76 | 685.90 | 2.92 | 2.99 | 3.12 | d-lactaldehyde dehydrogenase |
| 427649 | 55.84 | 407.30 | 534.42 | 227.69 | 2.87 | 3.26 | 2.03 | malate synthase |
| 51829 | 8.64 | 63.03 | 65.45 | 44.12 | 2.87 | 2.92 | 2.35 | malate dehydrogenase |
| 308008 | 9.45 | 68.85 | 107.64 | 53.25 | 2.87 | 3.51 | 2.49 | pyridoxamine 5 -phosphate oxidase- fmn-binding |
| 441328 | 153.79 | 1091.05 | 726.31 | 653.54 | 2.83 | 2.24 | 2.09 | acetyl- acetyl transferase |
| 157548 | 64.65 | 457.35 | 166.22 | 290.18 | 2.82 | 1.36 | 2.17 | nad-p-binding protein |
| 387282 | 22.62 | 157.59 | 839.07 | 121.43 | 2.80 | 5.21 | 2.42 | mboat-domain-containing protein |
| 32618 | 25.96 | 180.27 | 175.72 | 167.79 | 2.80 | 2.76 | 2.69 | sulfate permease |
| 439094 | 98.36 | 673.37 | 788.95 | 442.78 | 2.78 | 3.00 | 2.17 | class iii adh enzyme |
| 378893 | 68.40 | 459.34 | 358.64 | 310.22 | 2.75 | 2.39 | 2.18 | short-chain dehydrogenase reductase sdr |
| 243345 | 9.78 | 65.02 | 102.14 | 82.01 | 2.73 | 3.38 | 3.07 | proteophosphoglycan ppg4 |
| 436589 | 78.34 | 518.75 | 478.15 | 804.27 | 2.73 | 2.61 | 3.36 | cytochrome p450 oxidoreductase |
| 9148 | 10.06 | 65.72 | 41.73 | 28.90 | 2.71 | 2.05 | 1.52 | phytochrome-like protein |
| 477377 | 29.07 | 184.31 | 187.46 | 217.86 | 2.66 | 2.69 | 2.91 | carbon-nitrogen hydrolase |
| 322630 | 29.07 | 184.31 | 187.46 | 217.86 | 2.66 | 2.69 | 2.91 | c6 transcription factor |
| 411292 | 15.32 | 96.55 | 104.44 | 82.13 | 2.66 | 2.77 | 2.42 | alpha beta-hydrolase |
| 471015 | 38.87 | 240.06 | 353.42 | 179.65 | 2.63 | 3.18 | 2.21 | 3-oxoacid -transferase |
| 429046 | 51.61 | 314.86 | 381.76 | 203.53 | 2.61 | 2.89 | 1.98 | aromatic compound dioxygenase |
| 328638 | 3.41 | 20.81 | 21.26 | 28.57 | 2.61 | 2.64 | 3.07 | hypothetical protein HETIRDRAFT_328638 |
| 156301 | 161.54 | 969.68 | 806.25 | 1235.30 | 2.59 | 2.32 | 2.93 | hypothetical protein HETIRDRAFT_156301 |
| 315694 | 9.48 | 56.79 | 70.47 | 43.55 | 2.58 | 2.89 | 2.20 | c6 transcription factor |
| 419909 | 184.97 | 1099.83 | 910.06 | 811.02 | 2.57 | 2.30 | 2.13 | cytochrome p450 |
| 150932 | 171.25 | 997.45 | 548.48 | 577.27 | 2.54 | 1.68 | 1.75 | carbohydrate esterase family 4 protein |
| 242467 | 15.28 | 88.42 | 184.27 | 70.40 | 2.53 | 3.59 | 2.20 | fk506 suppressor |
| 48060 | 8.16 | 47.05 | 86.84 | 158.30 | 2.53 | 3.41 | 4.28 | amino acid transporter |
| 63265 | 39.55 | 227.10 | 1251.86 | 118.76 | 2.52 | 4.98 | 1.59 | glycosyltransferase family 8 protein |
| 173483 | 8.89 | 50.99 | 32.17 | 27.36 | 2.52 | 1.86 | 1.62 | hypothetical protein HETIRDRAFT_173483 |
| 470617 | 189.94 | 1065.55 | 732.44 | 438.52 | 2.49 | 1.95 | 1.21 | hypothetical protein HETIRDRAFT_470617 |
| 30755 | 84.52 | 472.52 | 272.51 | 299.84 | 2.48 | 1.69 | 1.83 | nad -binding protein |
| 468663 | 58.63 | 319.91 | 539.76 | 200.78 | 2.45 | 3.20 | 1.78 | 3-ketoacyl- thiolase |
| 436431 | 24.04 | 130.15 | 1195.89 | 99.69 | 2.44 | 5.64 | 2.05 | 2-cysteine peroxiredoxin |
| 311052 | 57.79 | 312.54 | 120.81 | 254.78 | 2.44 | 1.06 | 2.14 | crotonase |
| 320400 | 18.42 | 96.47 | 84.61 | 50.96 | 2.39 | 2.20 | 1.47 | #NAME? |
| 322274 | 20.25 | 105.54 | 111.65 | 106.15 | 2.38 | 2.46 | 2.39 | dihydropyrimidinase |
| 125840 | 11.69 | 60.61 | 76.17 | 62.33 | 2.37 | 2.70 | 2.41 | glycosyltransferase family 15 protein |
| 148791 | 2.76 | 14.20 | 33.68 | 36.67 | 2.37 | 3.61 | 3.73 | terpenoid synthase |
| 151380 | 6.89 | 35.39 | 116.86 | 155.81 | 2.36 | 4.08 | 4.50 | nad-p-binding protein |
| 62172 | 29.76 | 152.21 | 112.88 | 175.68 | 2.35 | 1.92 | 2.56 | cytochrome p450 |
| 428514 | 33.80 | 172.28 | 275.28 | 173.51 | 2.35 | 3.03 | 2.36 | hypothetical protein HETIRDRAFT_428514 |
| 328380 | 10.85 | 55.03 | 44.99 | 55.41 | 2.34 | 2.05 | 2.35 | icmt-domain-containing protein |
| 454399 | 460.24 | 2315.86 | 4831.11 | 1488.09 | 2.33 | 3.39 | 1.69 | aldehyde dehydrogenase |
| 235542 | 2.87 | 14.23 | 20.17 | 14.75 | 2.31 | 2.81 | 2.36 | hypothetical protein HETIRDRAFT_235542, partial |
| 381810 | 262.48 | 1302.15 | 880.65 | 1450.80 | 2.31 | 1.75 | 2.47 | s-adenosyl-l-methionine-dependent methyltransferase |
| 242526 | 5.43 | 26.92 | 22.47 | 15.99 | 2.31 | 2.05 | 1.56 | rna-binding domain-containing partial |
| 63331 | 166.13 | 807.05 | 1166.01 | 917.17 | 2.28 | 2.81 | 2.46 | multidrug resistance protein 4 |
| NA | 7.14 | 34.65 | 64.05 | 27.57 | 2.28 | 3.16 | 1.95 | secreted protein |
| 453755 | 66.36 | 319.70 | 286.22 | 196.79 | 2.27 | 2.11 | 1.57 | hypothetical protein HETIRDRAFT_453755 |
| 472133 | 266.73 | 1264.40 | 2678.31 | 1281.00 | 2.24 | 3.33 | 2.26 | class i glutamine amidotransferase-like protein |
| 468124 | 134.62 | 631.97 | 3821.51 | 536.60 | 2.23 | 4.83 | 1.99 | udp-glucose 4-epimerase |
| 53480 | 19.07 | 89.20 | 82.05 | 143.48 | 2.23 | 2.11 | 2.91 | acetyl- synthetase-like protein |
| 423519 | 29.33 | 135.21 | 210.94 | 118.31 | 2.20 | 2.85 | 2.01 | phenylalanine ammonia-lyase |
| 63784 | 30.82 | 141.72 | 133.77 | 91.98 | 2.20 | 2.12 | 1.58 | l-gulonolactone d-arabinono- -lactone oxidase |
| 56213 | 19.72 | 89.47 | 82.05 | 64.30 | 2.18 | 2.06 | 1.71 | amino acid permease |
| 478683 | 16.21 | 71.54 | 50.44 | 57.68 | 2.14 | 1.64 | 1.83 | pirin domain-containing protein |
| 151088 | 55.37 | 243.95 | 118.57 | 132.73 | 2.14 | 1.10 | 1.26 | six-hairpin glycosidase |
| 62868 | 43.58 | 188.35 | 321.81 | 352.31 | 2.11 | 2.88 | 3.02 | mfs general substrate transporter |
| 382866 | 15.37 | 65.18 | 150.39 | 118.00 | 2.08 | 3.29 | 2.94 | terpenoid synthase |
| 148835 | 9.62 | 40.50 | 93.73 | 50.15 | 2.07 | 3.28 | 2.38 | hypothetical protein HETIRDRAFT_148835 |
| 460318 | 23.47 | 98.60 | 251.69 | 83.11 | 2.07 | 3.42 | 1.82 | fad nad -binding domain-containing protein |
| 478862 | 105.68 | 440.74 | 652.10 | 1128.60 | 2.06 | 2.63 | 3.42 | hypothetical protein HETIRDRAFT_478862 |
| 480841 | 50.55 | 209.19 | 117.79 | 246.64 | 2.05 | 1.22 | 2.29 | aldo keto reductase |
| 120415 | 15.09 | 62.28 | 68.93 | 65.62 | 2.05 | 2.19 | 2.12 | hypothetical protein HETIRDRAFT_120415 |
| 305909 | 56.16 | 228.12 | 131.59 | 124.69 | 2.02 | 1.23 | 1.15 | peroxisomal membrane protein 4 |
| 441278 | 45.34 | 184.12 | 560.35 | 170.76 | 2.02 | 3.63 | 1.91 | hypothetical protein HETIRDRAFT_441278 |
| 380170 | 56.91 | 230.65 | 179.56 | 181.15 | 2.02 | 1.66 | 1.67 | glutamine synthetase guanido kinase |
| 32896 | 33.50 | 135.31 | 252.94 | 204.72 | 2.01 | 2.92 | 2.61 | nad-p-binding protein |
| 63309 | 36.60 | 145.97 | 122.26 | 124.34 | 2.00 | 1.74 | 1.76 | ---NA--- |
| 243211 | 10.52 | 41.94 | 46.19 | 41.45 | 2.00 | 2.13 | 1.98 | ph domain-containing protein |
| 326738 | 3.91 | 15.57 | 40.57 | 23.70 | 1.99 | 3.38 | 2.60 | indoleamine -dioxygenase |
| 383555 | 28.00 | 111.28 | 76.59 | 110.52 | 1.99 | 1.45 | 1.98 | hypothetical protein HETIRDRAFT_383555 |
| 39750 | 10.31 | 40.61 | 48.47 | 40.55 | 1.98 | 2.23 | 1.98 | carbohydrate esterase family 9 protein |
| 479927 | 23.34 | 91.40 | 115.85 | 96.44 | 1.97 | 2.31 | 2.05 | urea transporter |
| 469748 | 46.41 | 181.72 | 159.67 | 169.03 | 1.97 | 1.78 | 1.86 | #NAME? |
| 64605 | 36.96 | 144.69 | 542.71 | 133.81 | 1.97 | 3.88 | 1.86 | aspartic peptidase a1 |
| 121281 | 27.94 | 108.32 | 112.10 | 117.76 | 1.95 | 2.00 | 2.08 | choline ethanolaminephosphotransferase |
| 323280 | 217.91 | 843.77 | 1211.49 | 786.83 | 1.95 | 2.47 | 1.85 | hypothetical protein HETIRDRAFT_323280 |
| 456302 | 45.88 | 177.47 | 169.83 | 132.92 | 1.95 | 1.89 | 1.53 | hypothetical protein HETIRDRAFT_456302 |
| 155478 | 144.74 | 559.75 | 785.97 | 357.32 | 1.95 | 2.44 | 1.30 | proteasome-domain-containing protein |
| 452658 | 10.59 | 39.67 | 77.84 | 42.79 | 1.91 | 2.88 | 2.01 | hypothetical protein HETIRDRAFT_452658 |
| 50323 | 7.11 | 26.62 | 81.23 | 24.94 | 1.90 | 3.51 | 1.81 | fad nad p-binding domain-containing protein |
| 416223 | 6.56 | 24.50 | 16.75 | 16.88 | 1.90 | 1.35 | 1.36 | hypothetical protein HETIRDRAFT_416223 |
| 157553 | 55.00 | 205.49 | 160.75 | 144.24 | 1.90 | 1.55 | 1.39 | zinc-binding oxidoreductase |
| 63889 | 2.84 | 10.59 | 26.42 | 36.00 | 1.90 | 3.22 | 3.66 | alcohol oxidase |
| 471952 | 24.17 | 89.38 | 80.76 | 76.66 | 1.89 | 1.74 | 1.67 | urease accessory protein |
| 446181 | 56.64 | 208.80 | 245.76 | 219.22 | 1.88 | 2.12 | 1.95 | fmn-linked oxidoreductase |
| 40020 | 18.94 | 69.53 | 207.03 | 273.22 | 1.88 | 3.45 | 3.85 | peroxidase activity |
| 320909 | 15.67 | 57.22 | 100.43 | 59.55 | 1.87 | 2.68 | 1.93 | phenol 2-monooxygenase |
| 154851 | 39.27 | 143.23 | 153.91 | 183.38 | 1.87 | 1.97 | 2.22 | sulfate adenylyltransferase |
| 378419 | 173.53 | 627.29 | 950.62 | 609.70 | 1.85 | 2.45 | 1.81 | nadh-cytochrome b5 reductase |
| 125007 | 42.07 | 151.88 | 145.04 | 132.97 | 1.85 | 1.79 | 1.66 | hypothetical protein HETIRDRAFT_125007 |
| 162920 | 22.80 | 82.12 | 223.25 | 76.25 | 1.85 | 3.29 | 1.74 | high affinity methionine permease |
| 452497 | 18.26 | 65.74 | 75.72 | 56.13 | 1.85 | 2.05 | 1.62 | glycoside hydrolase family 63 protein |
| 157258 | 134.39 | 482.02 | 306.98 | 324.61 | 1.84 | 1.19 | 1.27 | peroxysomal citrate synthase |
| 330547 | 19.74 | 70.47 | 82.81 | 73.20 | 1.84 | 2.07 | 1.89 | 3-hydroxyanthranilic acid dioxygenase |
| 422466 | 26.12 | 91.80 | 672.71 | 130.54 | 1.81 | 4.69 | 2.32 | zinc-finger-containing protein |
| 53075 | 23.24 | 80.77 | 89.17 | 101.09 | 1.80 | 1.94 | 2.12 | mfs general substrate transporter |
| 121390 | 26.32 | 91.35 | 63.48 | 71.84 | 1.80 | 1.27 | 1.45 | het-c-domain-containing protein |
| 457014 | 11.59 | 40.20 | 28.92 | 34.80 | 1.79 | 1.32 | 1.59 | hypothetical protein HETIRDRAFT_457014 |
| 37098 | 25.01 | 86.72 | 113.19 | 176.81 | 1.79 | 2.18 | 2.82 | purine-cytosine permease fcy22 |
| 414892 | 121.45 | 420.26 | 481.15 | 273.51 | 1.79 | 1.99 | 1.17 | fructose- -bisphosphatase |
| 61142 | 18.98 | 64.01 | 134.50 | 103.65 | 1.75 | 2.82 | 2.45 | mfs polyamine transporter |
| 425696 | 98.85 | 333.26 | 203.55 | 283.43 | 1.75 | 1.04 | 1.52 | short-chain dehydrogenase |
| 35305 | 69.99 | 234.99 | 250.86 | 177.05 | 1.75 | 1.84 | 1.34 | hypothetical protein HETIRDRAFT_428372 |
| 413862 | 49.71 | 166.34 | 98.98 | 140.71 | 1.74 | 0.99 | 1.50 | hypothetical protein HETIRDRAFT_413862 |
| 143978 | 82.96 | 277.51 | 503.88 | 243.08 | 1.74 | 2.60 | 1.55 | hypothetical protein HETIRDRAFT_143978 |
| 147314 | 135.98 | 453.77 | 1001.75 | 548.59 | 1.74 | 2.88 | 2.01 | general amino acid permease 1 |
| 48385 | 19.44 | 64.24 | 49.94 | 60.00 | 1.72 | 1.36 | 1.63 | mitochondrial carrier |
| 166565 | 11.41 | 37.59 | 51.54 | 27.93 | 1.72 | 2.18 | 1.29 | abc1-domain-containing protein |
| 237830 | 4.41 | 14.38 | 17.63 | 14.23 | 1.71 | 2.00 | 1.69 | hypothetical protein HETIRDRAFT_237830, partial |
| 379532 | 132.20 | 422.32 | 362.60 | 361.68 | 1.68 | 1.46 | 1.45 | transketolase |
| 46561 | 10.52 | 33.53 | 99.90 | 38.11 | 1.67 | 3.25 | 1.86 | tpr-like protein |
| 32170 | 76.96 | 243.52 | 242.88 | 282.92 | 1.66 | 1.66 | 1.88 | ab-hydrolase |
| 245864 | 15.50 | 48.69 | 91.86 | 55.95 | 1.65 | 2.57 | 1.85 | transcription factor |
| 419288 | 22.70 | 71.22 | 65.52 | 58.37 | 1.65 | 1.53 | 1.36 | hypothetical protein HETIRDRAFT_419288 |
| 104547 | 29.71 | 92.25 | 89.87 | 137.55 | 1.63 | 1.60 | 2.21 | mfs general substrate transporter |
| 313519 | 36.54 | 112.59 | 97.95 | 97.01 | 1.62 | 1.42 | 1.41 | salicylate 1-monooxygenase |
| 461453 | 28.06 | 86.38 | 70.24 | 84.69 | 1.62 | 1.32 | 1.59 | hypothetical protein HETIRDRAFT_461453 |
| 65164 | 189.91 | 577.62 | 1229.49 | 659.19 | 1.60 | 2.69 | 1.80 | cytochrome p450 oxidoreductase |
| 440070 | 87.11 | 264.19 | 218.50 | 184.00 | 1.60 | 1.33 | 1.08 | inosine monophosphate dehydrogenase |
| 169836 | 222.01 | 667.99 | 1298.49 | 585.28 | 1.59 | 2.55 | 1.40 | cytochrome c peroxidase |
| 441637 | 24.85 | 74.56 | 79.03 | 51.97 | 1.59 | 1.67 | 1.06 | hypothetical protein HETIRDRAFT_441637 |
| 439243 | 31.61 | 94.42 | 134.62 | 80.52 | 1.58 | 2.09 | 1.35 | pkinase-domain-containing protein |
| 390687 | 15.90 | 47.00 | 72.06 | 104.99 | 1.56 | 2.18 | 2.72 | nadh:flavin oxidoreductase nadh oxidase |
| 305989 | 13.56 | 40.08 | 37.86 | 49.23 | 1.56 | 1.48 | 1.86 | glycine dehydrogenase |
| 330758 | 253.71 | 746.72 | 906.79 | 1198.06 | 1.56 | 1.84 | 2.24 | aldehyde dehydrogenase |
| 61201 | 20.02 | 58.75 | 71.33 | 71.38 | 1.55 | 1.83 | 1.83 | mfs general substrate transporter |
| 480101 | 16.16 | 47.27 | 57.13 | 37.55 | 1.55 | 1.82 | 1.22 | hypothetical protein HETIRDRAFT_480101 |
| 331537 | 30.55 | 89.25 | 109.56 | 132.86 | 1.55 | 1.84 | 2.12 | nad-p-binding protein |
| 153907 | 55.45 | 161.67 | 108.85 | 119.24 | 1.54 | 0.97 | 1.10 | general substrate transporter |
| 243856 | 93.39 | 268.86 | 1589.08 | 193.58 | 1.53 | 4.09 | 1.05 | hypothetical protein HETIRDRAFT_243856, partial |
| 119296 | 33.11 | 95.28 | 118.04 | 117.42 | 1.52 | 1.83 | 1.83 | hypothetical protein HETIRDRAFT_119296 |
| 65511 | 15.98 | 45.50 | 49.05 | 35.99 | 1.51 | 1.62 | 1.17 | fad nad-p-binding domain-containing protein |
| 236815 | 66.56 | 188.29 | 6299.68 | 283.95 | 1.50 | 6.56 | 2.09 | tetratricopeptide repeat protein 15-like |
| 156572 | 38.50 | 108.81 | 121.44 | 97.06 | 1.50 | 1.66 | 1.33 | kinase-like protein |
| 388721 | 60.49 | 170.26 | 373.93 | 177.70 | 1.49 | 2.63 | 1.55 | serine palmitoyltransferase 2 |
| 310226 | 38.79 | 108.84 | 225.26 | 80.25 | 1.49 | 2.54 | 1.05 | hypothetical protein HETIRDRAFT_310226 |
| 154946 | 48.33 | 132.74 | 102.51 | 116.76 | 1.46 | 1.08 | 1.27 | electron transfer flavo protein alpha subunit |
| 60087 | 31.75 | 86.90 | 266.37 | 103.39 | 1.45 | 3.07 | 1.70 | phospholipid-translocating p-type atpase |
| 154277 | 55.21 | 150.36 | 145.05 | 184.76 | 1.45 | 1.39 | 1.74 | pirin domain-containing protein |
| 406563 | 135.97 | 368.90 | 1141.35 | 319.30 | 1.44 | 3.07 | 1.23 | hypothetical protein HETIRDRAFT_406563 |
| 246773 | 51.53 | 139.02 | 119.11 | 149.93 | 1.43 | 1.21 | 1.54 | hypothetical protein HETIRDRAFT_246773, partial |
| 481508 | 106.88 | 287.32 | 1496.56 | 275.02 | 1.43 | 3.81 | 1.36 | fatty acid-2 hydroxylase |
| 321367 | 19.34 | 51.86 | 91.37 | 78.12 | 1.42 | 2.24 | 2.01 | hypothetical protein STEHIDRAFT_150846 |
| 146994 | 218.90 | 582.67 | 1191.81 | 502.11 | 1.41 | 2.44 | 1.20 | malate dehydrogenase |
| 450643 | 230.35 | 612.50 | 727.49 | 588.52 | 1.41 | 1.66 | 1.35 | citrate synthase |
| 388130 | 25.10 | 66.48 | 56.73 | 54.83 | 1.41 | 1.18 | 1.13 | hypothetical protein HETIRDRAFT_388130 |
| 169311 | 43.06 | 112.97 | 126.37 | 88.32 | 1.39 | 1.55 | 1.04 | kinase-like protein |
| 308224 | 28.67 | 74.93 | 76.34 | 92.91 | 1.39 | 1.41 | 1.70 | kinase-like protein |
| 108248 | 23.50 | 61.30 | 67.17 | 49.31 | 1.38 | 1.52 | 1.07 | hypothetical protein HETIRDRAFT_108248 |
| 315489 | 56.81 | 147.25 | 219.10 | 111.05 | 1.37 | 1.95 | 0.97 | enoyl- hydratase isomerase |
| 459487 | 14.29 | 37.02 | 303.44 | 33.89 | 1.37 | 4.41 | 1.25 | hypothetical protein HETIRDRAFT_459487 |
| 40642 | 15.42 | 39.69 | 54.99 | 81.94 | 1.36 | 1.83 | 2.41 | high-affinity nicotinic acid transporter |
| 318993 | 52.62 | 135.19 | 115.38 | 133.61 | 1.36 | 1.13 | 1.34 | hypothetical protein HETIRDRAFT_318993 |
| 476563 | 27.63 | 70.91 | 74.02 | 64.70 | 1.36 | 1.42 | 1.23 | quinate dehydrogenase |
| 445244 | 63.76 | 163.47 | 257.02 | 171.72 | 1.36 | 2.01 | 1.43 | hypothetical protein HETIRDRAFT_445244 |
| 432303 | 64.55 | 164.71 | 309.25 | 142.88 | 1.35 | 2.26 | 1.15 | v-type atpase |
| 439490 | 33.51 | 83.68 | 159.33 | 156.33 | 1.32 | 2.25 | 2.22 | mfs general substrate transporter |
| 444805 | 55.10 | 136.64 | 157.65 | 125.84 | 1.31 | 1.52 | 1.19 | duf500-domain-containing protein |
| 459332 | 93.76 | 231.75 | 189.49 | 232.03 | 1.31 | 1.02 | 1.31 | hypothetical protein HETIRDRAFT_459332 |
| 146597 | 129.26 | 319.23 | 306.84 | 256.43 | 1.30 | 1.25 | 0.99 | phospholipase carboxylesterase |
| 31013 | 85.57 | 208.26 | 226.61 | 216.45 | 1.28 | 1.41 | 1.34 | isopentenyldiphosphate isomerase |
| 459911 | 56.88 | 133.31 | 198.06 | 128.37 | 1.23 | 1.80 | 1.17 | hypothetical protein HETIRDRAFT_459911 |
| 46880 | 21.55 | 50.36 | 103.95 | 56.78 | 1.22 | 2.27 | 1.40 | ---NA--- |
| 65276 | 223.68 | 521.80 | 1302.57 | 506.55 | 1.22 | 2.54 | 1.18 | hypothetical protein HETIRDRAFT_65276 |
| 226882 | 16.94 | 39.34 | 44.71 | 37.02 | 1.22 | 1.40 | 1.13 | hypothetical protein HETIRDRAFT_226882, partial |
| 408174 | 131.80 | 302.28 | 372.65 | 332.76 | 1.20 | 1.50 | 1.34 | xylitol dehydrogenase |
| 331307 | 55.47 | 126.79 | 109.85 | 139.65 | 1.19 | 0.99 | 1.33 | hypothetical protein HETIRDRAFT_331307 |
| 380456 | 62.47 | 141.39 | 165.38 | 120.90 | 1.18 | 1.40 | 0.95 | arginase |
| 60007 | 225.99 | 508.96 | 489.58 | 544.30 | 1.17 | 1.12 | 1.27 | methionine adenosyltransferase |
| 418675 | 62.30 | 140.01 | 218.97 | 304.57 | 1.17 | 1.81 | 2.29 | heat shock protein 9 |
| 450852 | 15.61 | 34.60 | 36.00 | 34.97 | 1.15 | 1.21 | 1.16 | hypothetical protein HETIRDRAFT_450852 |
| 103055 | 33.56 | 73.63 | 188.22 | 74.62 | 1.13 | 2.49 | 1.15 | clathrin adaptor mu subunit |
| 471609 | 47.59 | 102.54 | 241.86 | 140.45 | 1.11 | 2.35 | 1.56 | alternative cyclin pcl1 |
| 453927 | 96.46 | 203.38 | 367.30 | 193.51 | 1.08 | 1.93 | 1.00 | fmn-linked oxidoreductase |
| 35598 | 74.28 | 155.79 | 196.82 | 143.56 | 1.07 | 1.41 | 0.95 | mitochondrial carrier |
| 455951 | 34.28 | 71.78 | 98.69 | 131.62 | 1.07 | 1.53 | 1.94 | urea transporter |
| 460734 | 30.64 | 64.04 | 273.00 | 62.75 | 1.06 | 3.16 | 1.03 | hypothetical protein HETIRDRAFT_460734 |
| 481946 | 24.36 | 50.88 | 64.59 | 74.07 | 1.06 | 1.41 | 1.60 | fad dependent oxidoreductase |
| 120862 | 15.78 | 32.82 | 52.52 | 36.90 | 1.06 | 1.73 | 1.23 | fad nad-p-binding domain-containing protein |
| 246490 | 31.69 | 65.68 | 65.85 | 84.18 | 1.05 | 1.06 | 1.41 | c2-domain-containing protein |
| 154894 | 68.54 | 141.26 | 140.30 | 142.26 | 1.04 | 1.03 | 1.05 | #NAME? |
| 241999 | 72.16 | 146.13 | 222.44 | 172.71 | 1.02 | 1.62 | 1.26 | hypothetical protein HETIRDRAFT_241999, partial |
| 442258 | 87.63 | 177.34 | 247.07 | 220.12 | 1.02 | 1.50 | 1.33 | glycine hydroxymethyltransferase |
| 391444 | 58.34 | 117.47 | 157.55 | 135.49 | 1.01 | 1.43 | 1.22 | fsh1-domain-containing protein |
| 242658 | 68.05 | 136.44 | 398.40 | 138.88 | 1.00 | 2.55 | 1.03 | orm1 type endoplasmic reticulum protein |
| 155103 | 21.36 | 42.53 | 47.70 | 47.57 | 0.99 | 1.16 | 1.15 | plp-dependent transferase |
| 146332 | 162.32 | 322.68 | 395.72 | 367.18 | 0.99 | 1.29 | 1.18 | regulatory protein cys-3 |
| 35715 | 132.17 | 261.93 | 315.11 | 295.94 | 0.99 | 1.25 | 1.16 | alpha beta-hydrolase |
| 145325 | 46.97 | 91.60 | 137.95 | 96.83 | 0.96 | 1.55 | 1.04 | mitochondrial inner membrane protein |

Table s7. List of CUGs specific to H. occidentale. L=liquid culture 2w, 4w and 6w=2weeks, 4weeks and 6weeks (Unit is FPKM), L-2w, -4w and-6w =liquid culture compare with 2weeks, 4weeks and 6weeks (unit is log2 fold change)

| JGI-ID | L | 2w | 4w | 6w | L-2w | L-4w | L-6w | SeqDesc |
| --- | --- | --- | --- | --- | --- | --- | --- | --- |
| 60203 | 39.90 | 117.23 | 86.11 | 95.07 | 1.55 | 1.11 | 1.25 | udp-galactose transporter |
| 308843 | 12.98 | 36.67 | 32.85 | 40.87 | 1.50 | 1.34 | 1.65 | phosphoglycerate mutase-like protein |
| 471495 | 125.25 | 337.78 | 308.69 | 293.13 | 1.43 | 1.30 | 1.23 | myo-inositol-1-phosphate synthase |
| 122126 | 0.47 | 13.49 | 5.03 | 4.78 | 4.85 | 3.43 | 3.35 | dak1-domain-containing protein |
| 415213 | 55.76 | 146.74 | 144.46 | 144.29 | 1.40 | 1.37 | 1.37 | hypothetical protein HETIRDRAFT_415213 |
|  | 2.79 | 19.14 | 26.85 | 23.23 | 2.78 | 3.27 | 3.06 | alpha beta-hydrolase |
|  | 41.65 | 79.45 | 154.66 | 121.91 | 0.93 | 1.89 | 1.55 | hypothetical protein HETIRDRAFT_472051 |
| 472051 | 11.18 | 21.09 | 36.35 | 35.92 | 0.92 | 1.70 | 1.68 | hypothetical protein HETIRDRAFT_472051 |
| 235489 | 2.24 | 8.96 | 6.29 | 7.76 | 2.00 | 1.49 | 1.79 | microtubule motor |
| 170468 | 53.44 | 678.39 | 347.97 | 223.29 | 3.67 | 2.70 | 2.06 | cytochrome p450 |
| 62665 | 7.78 | 175.52 | 109.70 | 87.16 | 4.50 | 3.82 | 3.49 | glycoside hydrolase family 2 protein |
| 167279 | 8.06 | 29.09 | 28.79 | 27.06 | 1.85 | 1.84 | 1.75 | hypothetical protein HETIRDRAFT_167279 |
|  | 25.31 | 53.02 | 48.95 | 46.91 | 1.07 | 0.95 | 0.89 | hypothetical protein HETIRDRAFT_244598 |
| 41157 | 4.25 | 9.36 | 8.99 | 9.03 | 1.14 | 1.08 | 1.09 | spoc domain-like protein |
|  | 7.93 | 380.50 | 51.55 | 50.82 | 5.59 | 2.70 | 2.68 | hypothetical protein HETIRDRAFT_305492 |
| 306992 | 10.97 | 30.67 | 36.15 | 36.09 | 1.48 | 1.72 | 1.72 | fmn-linked oxidoreductase |
| 305254 | 3.53 | 9.57 | 7.28 | 9.44 | 1.44 | 1.04 | 1.42 | kinesin-domain-containing protein |
| 457097 | 3.63 | 8.92 | 9.02 | 8.31 | 1.30 | 1.31 | 1.20 | kinase-like protein |
| 436826 | 10.44 | 42.05 | 36.36 | 23.84 | 2.01 | 1.80 | 1.19 | hypothetical protein HETIRDRAFT_436826 |
| 66331 | 14.10 | 29.64 | 35.55 | 34.45 | 1.07 | 1.33 | 1.29 | g-alpha-domain-containing protein |
| 329337 | 36.89 | 207.92 | 113.35 | 129.12 | 2.49 | 1.62 | 1.81 | serine carboxypeptidase |
| 481260 | 37.54 | 95.63 | 71.77 | 76.22 | 1.35 | 0.94 | 1.02 | hypothetical protein STEHIDRAFT_65725 |
|  | 6.77 | 26.45 | 25.83 | 17.23 | 1.97 | 1.93 | 1.35 | hypothetical protein HETIRDRAFT_412107 |
| 125898 | 26.74 | 175.78 | 234.49 | 304.66 | 2.72 | 3.13 | 3.51 | cytochrome p450 |
| 435740 | 12.61 | 426.14 | 152.99 | 176.24 | 5.08 | 3.60 | 3.80 | mandelate racemase muconate lactonizing enzyme |
| 67107 | 2.05 | 623.37 | 172.14 | 230.08 | 8.25 | 6.39 | 6.81 | general substrate transporter |
| 325916 | 93.66 | 292.46 | 363.60 | 272.48 | 1.64 | 1.96 | 1.54 | duf706-domain-containing protein |
| 157315 | 7.85 | 38.67 | 21.87 | 27.00 | 2.30 | 1.48 | 1.78 | pkinase-domain-containing protein |
| 441380 | 9.06 | 18.72 | 19.38 | 20.89 | 1.05 | 1.10 | 1.20 | zinc c3hc4 type (ring finger) protein |
| 12581 | 17.65 | 52.27 | 57.26 | 34.81 | 1.57 | 1.70 | 0.98 | mfs sugar partial |
|  | 2.46 | 10.63 | 5.69 | 7.57 | 2.11 | 1.21 | 1.62 | pin domain-like protein |
| 411170 | 37.66 | 223.85 | 136.13 | 150.58 | 2.57 | 1.85 | 2.00 | #NAME? |
| 420741 | 14.01 | 163.92 | 63.45 | 71.12 | 3.55 | 2.18 | 2.34 | transmembrane protein |
| 320693 | 1.91 | 6.06 | 4.86 | 5.66 | 1.66 | 1.35 | 1.57 | hypothetical protein HETIRDRAFT_320693 |
| 61998 | 5.86 | 82.91 | 79.14 | 41.16 | 3.82 | 3.75 | 2.81 | mfs sugar transporter |
| 427781 | 17.70 | 50.30 | 43.56 | 59.08 | 1.51 | 1.30 | 1.74 | nucleoside hydrolase |
| 61954 | 26.31 | 86.20 | 110.88 | 66.06 | 1.71 | 2.08 | 1.33 | subtilisin-like protease |
| 434783 | 4.91 | 123.45 | 211.08 | 329.71 | 4.65 | 5.43 | 6.07 | #NAME? |
| 434768 | 2.04 | 92.73 | 34.51 | 29.71 | 5.50 | 4.08 | 3.86 | dihydroxy-acid dehydratase |
| 46597 | 7.98 | 79.55 | 21.15 | 27.48 | 3.32 | 1.41 | 1.78 | glycoside hydrolase family 3 protein |
| 155493 | 41.81 | 198.60 | 104.56 | 109.19 | 2.25 | 1.32 | 1.39 | alcohol dehydrogenase |
| 427098 | 9.66 | 24.49 | 23.19 | 20.96 | 1.34 | 1.26 | 1.12 | hypothetical protein HETIRDRAFT_427098 |
| 451528 | 1.56 | 8.15 | 4.36 | 5.39 | 2.38 | 1.48 | 1.79 | hypothetical protein HETIRDRAFT_451528 |
| 459074 | 54.99 | 1074.23 | 821.16 | 1153.08 | 4.29 | 3.90 | 4.39 | protein |
| 451607 | 18.00 | 276.53 | 283.39 | 126.68 | 3.94 | 3.98 | 2.81 | had-like protein |
| 317220 | 23.75 | 43.90 | 49.11 | 46.55 | 0.89 | 1.05 | 0.97 | mfs general substrate transporter |
| 331340 | 7.79 | 182.15 | 18.08 | 24.57 | 4.55 | 1.21 | 1.66 | endo- -beta-xylanase |
|  | 35.43 | 380.67 | 126.08 | 183.96 | 3.43 | 1.83 | 2.38 | s-adenosyl-l-methionine-dependent methyltransferase |
| 323246 | 4.72 | 14.49 | 15.46 | 15.70 | 1.62 | 1.71 | 1.73 | hypothetical protein HETIRDRAFT_323246 |
| 240447 | 13.49 | 29.29 | 27.25 | 28.84 | 1.12 | 1.01 | 1.10 | p-loop containing nucleoside triphosphate hydrolase |
| 47201 | 6.69 | 34.99 | 16.37 | 20.62 | 2.39 | 1.29 | 1.62 | hypothetical protein HETIRDRAFT_47201 |
| 155557 | 3.74 | 184.26 | 42.55 | 67.49 | 5.62 | 3.51 | 4.17 | mfs general substrate transporter |
|  | 1.20 | 108.17 | 40.23 | 52.89 | 6.50 | 5.07 | 5.46 | glycoside hydrolase family 105 protein |
| 439197 | 1.25 | 3.91 | 3.04 | 3.43 | 1.64 | 1.28 | 1.46 | Cell differentiation regulator of the Headcase family |
| 313554 | 66.58 | 132.90 | 155.82 | 169.56 | 1.00 | 1.23 | 1.35 | hypothetical protein HETIRDRAFT_313540 |
| 124013 | 17.04 | 67.37 | 47.07 | 34.11 | 1.98 | 1.47 | 1.00 | fatty acid hydroxylase |
| 169178 | 12.46 | 28.09 | 28.56 | 31.46 | 1.17 | 1.20 | 1.34 | homeobox-domain-containing protein |
| 459262 | 20.41 | 69.96 | 70.99 | 74.13 | 1.78 | 1.80 | 1.86 | hypothetical protein HETIRDRAFT_459262 |
| 50956 | 3.23 | 1355.86 | 91.90 | 92.11 | 8.71 | 4.83 | 4.83 | glycoside hydrolase family 79 protein |
| 308216 | 76.12 | 300.73 | 205.38 | 228.54 | 1.98 | 1.43 | 1.59 | pr-1-like protein |
| 424240 | 9.07 | 43.24 | 38.47 | 39.77 | 2.25 | 2.08 | 2.13 | hypothetical protein HETIRDRAFT_424240 |
| 306187 | 16.63 | 29.39 | 40.52 | 31.37 | 0.82 | 1.28 | 0.92 | hypothetical protein HETIRDRAFT_306187 |
| 59470 | 17.56 | 46.68 | 47.85 | 52.59 | 1.41 | 1.45 | 1.58 | carbon-nitrogen hydrolase |
| 30690 | 51.77 | 152.68 | 165.19 | 186.90 | 1.56 | 1.67 | 1.85 | carbohydrate esterase family 1 protein |
|  | 4.89 | 14.68 | 15.04 | 15.33 | 1.59 | 1.62 | 1.65 | hypothetical protein STEHIDRAFT_70790 |
| 468777 | 48.80 | 132.70 | 104.66 | 96.26 | 1.44 | 1.10 | 0.98 | clavaminate synthase-like protein |
| 59016 | 57.09 | 245.09 | 137.50 | 137.24 | 2.10 | 1.27 | 1.27 | aldo keto reductase |
| 435195 | 43.11 | 119.74 | 139.23 | 145.82 | 1.47 | 1.69 | 1.76 | acyl- oxidase |
| 322707 | 13.27 | 53.10 | 32.09 | 40.52 | 2.00 | 1.27 | 1.61 | hypothetical transcription factor |
| 322640 | 2.68 | 6.79 | 7.11 | 6.09 | 1.34 | 1.41 | 1.18 | dna replication atp-dependent helicase dna2 |
| 150132 | 4.40 | 12.67 | 11.67 | 14.50 | 1.53 | 1.41 | 1.72 | gamma tubulin |
|  | 9.54 | 28.02 | 20.32 | 27.22 | 1.55 | 1.09 | 1.51 | 3-hydroxyisobutyrate dehydrogenase |
| 44672 | 51.61 | 96.42 | 124.86 | 94.42 | 0.90 | 1.27 | 0.87 | far-17a aig1-like protein |
| 427527 | 7.30 | 16.00 | 17.02 | 15.39 | 1.13 | 1.22 | 1.08 | hypothetical protein HETIRDRAFT_427527 |
| 46540 | 6.00 | 12.42 | 11.95 | 16.30 | 1.05 | 0.99 | 1.44 | #NAME? |
| 33452 | 39.63 | 133.11 | 98.89 | 96.27 | 1.75 | 1.32 | 1.28 | glycoside hydrolase family 23 protein |
| 12392 | 0.64 | 267.56 | 13.75 | 15.47 | 8.70 | 4.42 | 4.59 | glycoside hydrolase family 28 protein |
| 431393 | 9.78 | 342.05 | 73.48 | 66.48 | 5.13 | 2.91 | 2.76 | proteasome ( macropain) activator subunit 3 |
|  | 11.44 | 78.41 | 79.00 | 107.07 | 2.78 | 2.79 | 3.23 | hypothetical protein GLOTRDRAFT_21993 |
| 58832 | 12.28 | 58.23 | 65.16 | 78.75 | 2.25 | 2.41 | 2.68 | mfs general substrate transporter |
| 42460 | 2.49 | 7.74 | 6.38 | 7.48 | 1.64 | 1.36 | 1.59 | dna repair protein rad5 |
| 321174 | 12.10 | 24.15 | 38.82 | 26.21 | 1.00 | 1.68 | 1.11 | hypothetical protein STEHIDRAFT_171190 |
| 410013 | 2.56 | 22.13 | 24.00 | 27.67 | 3.11 | 3.23 | 3.43 | hypothetical protein HETIRDRAFT_410013 |
| 319471 | 44.49 | 214.86 | 122.13 | 104.25 | 2.27 | 1.46 | 1.23 | hypothetical protein HETIRDRAFT_319471 |
| 319541 | 4.36 | 11.30 | 20.47 | 10.29 | 1.37 | 2.23 | 1.24 | hypothetical protein HETIRDRAFT_319541 |
| 410548 | 21.15 | 47.71 | 42.68 | 45.00 | 1.17 | 1.01 | 1.09 | hypothetical protein HETIRDRAFT_410548 |
|  | 7.41 | 93.58 | 127.53 | 99.03 | 3.66 | 4.10 | 3.74 | cytochrome p450 |
| 434345 | 1.36 | 24.99 | 66.36 | 30.17 | 4.20 | 5.61 | 4.47 | non-catalytic module family expn protein |
| 384428 | 12.96 | 383.98 | 88.85 | 56.81 | 4.89 | 2.78 | 2.13 | di-copper centre-containing protein |
|  | 22.46 | 261.45 | 147.36 | 144.44 | 3.54 | 2.71 | 2.68 | hypothetical protein HETIRDRAFT_319025 |
| 37715 | 0.90 | 224.55 | 16.58 | 19.91 | 7.96 | 4.20 | 4.47 | arabinogalactan endo- -beta-galactosidase |
| 106092 | 1.34 | 189.59 | 21.68 | 62.15 | 7.14 | 4.01 | 5.53 | 3-oxoacyl-reductase 1 |
| 480809 | 32.34 | 95.41 | 154.94 | 160.43 | 1.56 | 2.26 | 2.31 | glucooligosaccharide oxidase |
|  | 9.04 | 27.73 | 23.63 | 23.85 | 1.62 | 1.39 | 1.40 | hypothetical protein HETIRDRAFT_446573 |
| 157934 | 8.93 | 99.34 | 32.86 | 45.16 | 3.48 | 1.88 | 2.34 | sugar transporter |
| 241554 | 34.17 | 112.00 | 78.89 | 69.36 | 1.71 | 1.21 | 1.02 | hypothetical protein HETIRDRAFT_241554 |
| 383456 | 62.40 | 146.01 | 147.89 | 120.33 | 1.23 | 1.24 | 0.95 | tumor-related protein |
| 54732 | 8.48 | 35.20 | 23.93 | 25.00 | 2.05 | 1.50 | 1.56 | Choline dehydrogenase and related flavoproteins |
| 55075 | 6.18 | 30.51 | 19.76 | 21.35 | 2.30 | 1.68 | 1.79 | gtpase binding protein rid1 |
| 452487 | 15.67 | 124.49 | 32.91 | 46.41 | 2.99 | 1.07 | 1.57 | secreted protein |
| 434918 | 18.86 | 37.30 | 40.01 | 39.14 | 0.98 | 1.09 | 1.05 | duf803-domain-containing protein |
|  | 6.87 | 30.53 | 17.87 | 20.36 | 2.15 | 1.38 | 1.57 | hypothetical protein HETIRDRAFT_440636 |
| 125080 | 7.73 | 42.76 | 17.12 | 17.85 | 2.47 | 1.15 | 1.21 | aromatic compound dioxygenase |
| 51894 | 8.87 | 42.29 | 42.17 | 31.64 | 2.25 | 2.25 | 1.84 | aquaporin |
| 434148 | 53.67 | 189.82 | 121.53 | 154.97 | 1.82 | 1.18 | 1.53 | protein |
|  | 10.25 | 378.38 | 78.69 | 115.12 | 5.21 | 2.94 | 3.49 | hypothetical protein HETIRDRAFT_141440 |
| 457384 | 7.34 | 13.85 | 19.56 | 17.36 | 0.92 | 1.41 | 1.24 | hypothetical protein HETIRDRAFT_457384 |
| 327342 | 5.17 | 25.79 | 19.13 | 17.86 | 2.32 | 1.89 | 1.79 | alpha beta-hydrolase |
| 479179 | 8.42 | 19.16 | 19.84 | 22.40 | 1.19 | 1.24 | 1.41 | kinase-like protein |
| 444537 | 4.53 | 13.40 | 37.27 | 90.26 | 1.57 | 3.04 | 4.32 | hypothetical protein HETIRDRAFT_458567 |
| 157171 | 22.66 | 410.58 | 68.94 | 65.29 | 4.18 | 1.61 | 1.53 | galactan -beta-galactosidase |
| 315222 | 82.81 | 1471.88 | 746.77 | 998.76 | 4.15 | 3.17 | 3.59 | hypothetical protein HETIRDRAFT_315222 |
| 124425 | 12.99 | 173.30 | 176.57 | 136.64 | 3.74 | 3.76 | 3.39 | cytochrome p450 |
|  | 32.27 | 163.97 | 71.52 | 81.56 | 2.35 | 1.15 | 1.34 | cytochrome p450 |
| 452835 | 6.54 | 17.37 | 22.94 | 33.46 | 1.41 | 1.81 | 2.36 | protein |
|  | 19.43 | 389.85 | 206.65 | 266.02 | 4.33 | 3.41 | 3.78 | hypothetical protein HETIRDRAFT_424707 |
| 379299 | 4.66 | 13.00 | 11.85 | 16.50 | 1.48 | 1.35 | 1.82 | fad nad p-binding domain-containing protein |
| 154589 | 212.25 | 838.76 | 1583.13 | 1128.09 | 1.98 | 2.90 | 2.41 | #NAME? |
| 438158 | 7.27 | 64.78 | 41.31 | 86.60 | 3.15 | 2.51 | 3.57 | hypothetical protein HETIRDRAFT_438158 |
| 168564 | 14.04 | 50.74 | 29.44 | 27.75 | 1.85 | 1.07 | 0.98 | wsc domain-containing protein |
|  | 8.99 | 27.10 | 52.65 | 42.62 | 1.59 | 2.55 | 2.24 | doda_amamu ame: full=dopa -dioxygenase |
| 430858 | 11.43 | 781.07 | 287.68 | 279.86 | 6.09 | 4.65 | 4.61 | hypothetical protein HETIRDRAFT_430858 |
| 482211 | 6.73 | 20.69 | 18.51 | 15.54 | 1.62 | 1.46 | 1.21 | hypothetical protein HETIRDRAFT_482211 |
| 426546 | 4.73 | 22.49 | 23.67 | 17.78 | 2.25 | 2.32 | 1.91 | aminoglycoside phosphotransferase |
| 123490 | 14.95 | 63.53 | 28.98 | 34.36 | 2.09 | 0.96 | 1.20 | glycoside hydrolase family 3 protein |
|  | 27.38 | 51.49 | 53.46 | 62.95 | 0.91 | 0.97 | 1.20 | hypothetical protein HETIRDRAFT_415457 |
| 102391 | 10.03 | 115.58 | 35.91 | 66.66 | 3.53 | 1.84 | 2.73 | alpha beta-hydrolase |
| 34134 | 25.54 | 95.78 | 75.07 | 63.57 | 1.91 | 1.56 | 1.32 | aldehyde dehydrogenase |
| 415481 | 7.24 | 90.07 | 45.48 | 29.55 | 3.64 | 2.65 | 2.03 | hypothetical protein HETIRDRAFT_415481 |
| 415334 | 11.88 | 51.81 | 71.83 | 62.80 | 2.12 | 2.60 | 2.40 | hypothetical protein HETIRDRAFT_415334 |
| 58645 | 4.15 | 9.54 | 8.32 | 10.01 | 1.20 | 1.00 | 1.27 | smc protein |
| 479833 | 11.45 | 26.81 | 46.29 | 40.38 | 1.23 | 2.02 | 1.82 | hypothetical protein HETIRDRAFT_479833 |
| 65589 | 4.57 | 55.29 | 31.78 | 45.89 | 3.60 | 2.80 | 3.33 | cytochrome p450 |
| 436220 | 4.60 | 12.11 | 9.77 | 10.67 | 1.40 | 1.09 | 1.21 | von Willebrand factor |
| 238463 | 0.66 | 23.04 | 3.17 | 3.11 | 5.12 | 2.26 | 2.23 | hypothetical protein HETIRDRAFT_238463 |
| 156364 | 1.25 | 1194.30 | 65.87 | 91.82 | 9.89 | 5.71 | 6.19 | polysaccharide lyase family 4 protein |
| 242372 | 6.76 | 57.86 | 22.97 | 23.47 | 3.10 | 1.76 | 1.80 | hypothetical protein HETIRDRAFT_242372 |
| 107260 | 6.34 | 434.09 | 30.94 | 36.36 | 6.10 | 2.29 | 2.52 | carbohydrate esterase family 12 protein |
| 315249 | 14.14 | 41.43 | 26.46 | 31.63 | 1.55 | 0.90 | 1.16 | g2 mitotic-specific cyclin-b |
| 148544 | 61.51 | 151.42 | 113.21 | 114.52 | 1.30 | 0.88 | 0.90 | alcohol dehydrogenase |
| 54628 | 3.14 | 9.64 | 8.80 | 9.51 | 1.62 | 1.49 | 1.60 | bub protein kinase |
| 435671 | 25.88 | 54.46 | 51.51 | 63.74 | 1.07 | 0.99 | 1.30 | gata-4 5 6 transcription factor |
| 58715 | 35.79 | 177.57 | 107.78 | 130.85 | 2.31 | 1.59 | 1.87 | aldo keto reductase |
| 413123 | 13.29 | 71.60 | 54.77 | 61.65 | 2.43 | 2.04 | 2.21 | enoyl- hydratase carnithine racemase |
| 237994 | 5.73 | 12.61 | 15.69 | 13.88 | 1.14 | 1.45 | 1.28 | protein |
| 105309 | 1.32 | 5.45 | 3.58 | 4.15 | 2.05 | 1.44 | 1.65 | timeless-domain-containing protein |
| 327719 | 19.55 | 50.80 | 41.49 | 41.10 | 1.38 | 1.09 | 1.07 | ---NA--- |
| 65373 | 4.05 | 125.73 | 8.97 | 9.43 | 4.95 | 1.15 | 1.22 | glycoside hydrolase family 31 protein |
|  | 13.79 | 34.45 | 54.98 | 67.12 | 1.32 | 2.00 | 2.28 | hypothetical protein HETIRDRAFT_106182 |
| 446593 | 17.52 | 83.37 | 65.56 | 74.64 | 2.25 | 1.90 | 2.09 | homeobox-domain-containing protein |
| 454145 | 1.33 | 4.58 | 5.59 | 6.92 | 1.79 | 2.08 | 2.39 | alpha beta-hydrolase |
| 312776 | 16.88 | 50.31 | 44.21 | 42.71 | 1.58 | 1.39 | 1.34 | hypothetical protein HETIRDRAFT_312776 |
| 436850 | 6.90 | 32.93 | 17.01 | 20.28 | 2.26 | 1.30 | 1.56 | acetyl- synthetase-like protein |
| 106581 | 4.40 | 13.27 | 15.31 | 15.27 | 1.59 | 1.80 | 1.79 | abc partial |
| 162730 | 5.05 | 524.34 | 29.06 | 35.22 | 6.70 | 2.52 | 2.80 | glycoside hydrolase family 51 protein |
|  | 17.32 | 508.43 | 94.86 | 84.76 | 4.88 | 2.45 | 2.29 | glycoside hydrolase family 51 protein |
| 53076 | 14.68 | 99.47 | 45.79 | 51.94 | 2.76 | 1.64 | 1.82 | glycoside hydrolase family 5 protein |
| 45732 | 4.75 | 597.71 | 26.14 | 27.96 | 6.98 | 2.46 | 2.56 | carbohydrate esterase family 8 protein |
| 33073 | 21.95 | 91.85 | 45.11 | 57.97 | 2.07 | 1.04 | 1.40 | glycoside hydrolase family 20 protein |
| 168848 | 8.08 | 20.32 | 21.55 | 22.63 | 1.33 | 1.41 | 1.49 | potassium transporter |
| 427315 | 21.57 | 51.60 | 60.30 | 42.73 | 1.26 | 1.48 | 0.99 | #NAME? |
|  | 2.19 | 12.87 | 11.11 | 22.79 | 2.56 | 2.34 | 3.38 | hypothetical protein M422DRAFT_212789 |
|  | 7.46 | 22.96 | 16.71 | 20.04 | 1.62 | 1.16 | 1.42 | phenylacetyl- ligase |
| 409794 | 8.51 | 17.06 | 25.33 | 22.13 | 1.00 | 1.57 | 1.38 | acid protease |
| 310350 | 12.22 | 23.68 | 23.46 | 34.02 | 0.95 | 0.94 | 1.48 | glycoside hydrolase family 5 protein |
| 309196 | 7.05 | 305.86 | 75.46 | 94.29 | 5.44 | 3.42 | 3.74 | fad nad-p-binding domain-containing protein |
| 27192 | 0.64 | 376.53 | 10.77 | 180.46 | 9.20 | 4.07 | 8.13 | hydrophobic surface binding protein |
| 152014 | 3.75 | 479.68 | 20.30 | 26.82 | 7.00 | 2.43 | 2.84 | endo-polygalacturonase pg1 |
| 322477 | 5.03 | 46.27 | 13.26 | 17.05 | 3.20 | 1.40 | 1.76 | ---NA--- |
|  | 26.60 | 53.47 | 49.89 | 56.63 | 1.01 | 0.91 | 1.09 | hypothetical protein HETIRDRAFT_439473 |
| 382802 | 4.63 | 58.14 | 19.41 | 18.96 | 3.65 | 2.07 | 2.04 | terpenoid synthase |
| 105890 | 1.73 | 254.63 | 41.50 | 60.41 | 7.20 | 4.59 | 5.13 | lignin expressed protein lep1 |
| 480543 | 3.33 | 7.10 | 7.52 | 7.61 | 1.09 | 1.17 | 1.19 | piwi-domain-containing protein |
| 386078 | 1.68 | 6.27 | 6.83 | 5.78 | 1.89 | 2.02 | 1.78 | hypothetical protein HETIRDRAFT_386078 |
| 11764 | 6.37 | 28.35 | 33.06 | 24.47 | 2.15 | 2.38 | 1.94 | amine oxidase protein |
| 408734 | 9.81 | 55.60 | 98.79 | 68.23 | 2.50 | 3.33 | 2.80 | hypothetical protein HETIRDRAFT_408734 |
|  | 35.25 | 90.37 | 95.04 | 104.22 | 1.36 | 1.43 | 1.56 | cral trio domain-containing protein |
| 99000 | 5.23 | 14.36 | 12.24 | 22.34 | 1.46 | 1.23 | 2.10 | mfs general substrate transporter |
|  | 6.71 | 21.56 | 24.34 | 28.41 | 1.68 | 1.86 | 2.08 | fad nad-binding domain-containing protein |
| 58532 | 6.71 | 21.56 | 24.34 | 28.41 | 1.68 | 1.86 | 2.08 | fad nad-binding domain-containing protein |
| 104169 | 7.21 | 173.96 | 74.83 | 99.47 | 4.59 | 3.37 | 3.79 | class 3 |
| 155952 | 3.74 | 65.85 | 24.12 | 40.51 | 4.14 | 2.69 | 3.44 | aaa atpase |
| 104184 | 2.53 | 19.93 | 22.71 | 14.82 | 2.98 | 3.17 | 2.55 | glycoside hydrolase family 18 protein |
| 171713 | 7.64 | 25.31 | 18.04 | 21.21 | 1.73 | 1.24 | 1.47 | protein |
| 63728 | 31.06 | 94.39 | 76.69 | 83.05 | 1.60 | 1.30 | 1.42 | ncs1 nucleoside transporter |
|  | 5.73 | 30.84 | 20.42 | 22.19 | 2.43 | 1.83 | 1.95 | arad-like aldolase epimerase |
| 103610 | 2.53 | 17.44 | 24.93 | 21.09 | 2.78 | 3.30 | 3.06 | hypothetical protein HETIRDRAFT_103610 |
| 68421 | 58.46 | 331.79 | 330.16 | 337.24 | 2.50 | 2.50 | 2.53 | gpr1 family protein |
| 325829 | 9.84 | 31.25 | 28.14 | 19.57 | 1.67 | 1.52 | 0.99 | crotonase |
| 451140 | 3.96 | 17.90 | 9.66 | 14.41 | 2.18 | 1.29 | 1.86 | kinase-like protein |
|  | 11.85 | 198.02 | 349.71 | 267.44 | 4.06 | 4.88 | 4.50 | hypothetical iron reductase |
| 36460 | 19.81 | 376.23 | 622.47 | 473.54 | 4.25 | 4.97 | 4.58 | ferric reductase |
| 423596 | 1.69 | 8.98 | 5.39 | 5.61 | 2.41 | 1.67 | 1.73 | hypothetical protein HETIRDRAFT_423596 |
| 57041 | 17.11 | 66.08 | 66.27 | 68.74 | 1.95 | 1.95 | 2.01 | phospholipid diacylglycerol acyltransferase |
| 319510 | 11.51 | 27.34 | 27.32 | 29.68 | 1.25 | 1.25 | 1.37 | thymidylate synthase |
|  | 7.71 | 47.04 | 24.28 | 19.12 | 2.61 | 1.66 | 1.31 | fad-binding domain-containing protein |
| 147230 | 10.73 | 40.58 | 28.54 | 37.38 | 1.92 | 1.41 | 1.80 | nicotinamide mononucleotide permease |
| 453203 | 12.60 | 57.09 | 50.31 | 60.20 | 2.18 | 2.00 | 2.26 | aldolase |
| 103957 | 5.04 | 40.68 | 50.27 | 66.03 | 3.01 | 3.32 | 3.71 | nucleoside-diphosphate-sugar epimerase |
| 105500 | 29.79 | 113.73 | 75.59 | 86.36 | 1.93 | 1.34 | 1.54 | hypothetical protein HETIRDRAFT_105500 |
| 445737 | 1.09 | 9.58 | 6.76 | 6.11 | 3.14 | 2.63 | 2.49 | glycoside hydrolase family 47 protein |
| 459941 | 8.49 | 186.05 | 55.21 | 108.46 | 4.45 | 2.70 | 3.68 | glycerol kinase |
| 121313 | 1.94 | 46.55 | 7.37 | 6.28 | 4.58 | 1.92 | 1.69 | glycoside hydrolase family 28 protein |
| 155612 | 6.16 | 76.27 | 24.59 | 41.11 | 3.63 | 2.00 | 2.74 | Glycoside Hydrolase Family 78 proteinor 37 |
| 63436 | 24.81 | 59.38 | 52.89 | 51.40 | 1.26 | 1.09 | 1.05 | mfs general substrate transporter |
| 37034 | 3.49 | 24.17 | 48.58 | 49.31 | 2.79 | 3.80 | 3.82 | mfs general substrate transporter |
| 480313 | 117.59 | 356.40 | 350.05 | 397.10 | 1.60 | 1.57 | 1.76 | peroxisomal dehydratase |
|  | 7.07 | 31.10 | 134.02 | 71.68 | 2.14 | 4.25 | 3.34 | cytochrome p450 |
|  | 10.55 | 57.13 | 182.28 | 101.80 | 2.44 | 4.11 | 3.27 | cytochrome p450 |
| 156196 | 13.49 | 35.80 | 32.10 | 31.98 | 1.41 | 1.25 | 1.25 | K+/H+ antiporter |
| 51381 | 8.11 | 35.22 | 28.77 | 29.38 | 2.12 | 1.83 | 1.86 | filamentous muscle protein titin- related protein |
|  | 29.02 | 88.59 | 110.26 | 85.97 | 1.61 | 1.93 | 1.57 | fruit-body specific gene c |
|  | 0.78 | 13.64 | 10.64 | 11.76 | 4.14 | 3.78 | 3.92 | cytochrome p450 |
| 104017 | 1.59 | 11.07 | 5.13 | 4.07 | 2.80 | 1.69 | 1.36 | glycoside hydrolase family 29 protein |
| 451904 | 4.24 | 9.81 | 23.95 | 17.41 | 1.21 | 2.50 | 2.04 | duf917-domain-containing protein |
| 64723 | 3.23 | 36.12 | 12.60 | 9.79 | 3.48 | 1.96 | 1.60 | esterase lipase thioesterase |
| 389494 | 14.36 | 32.31 | 44.55 | 42.93 | 1.17 | 1.63 | 1.58 | cbf-domain-containing protein |
| 313664 | 5.33 | 15.37 | 10.85 | 12.03 | 1.53 | 1.02 | 1.17 | hd-domain pdease-like protein |
| 453187 | 1.87 | 9.00 | 10.33 | 9.23 | 2.26 | 2.46 | 2.30 | hypothetical protein HETIRDRAFT_453187 |
| 44857 | 25.93 | 241.70 | 326.61 | 368.21 | 3.22 | 3.65 | 3.83 | glutathione-independent formaldehyde dehydrogenase |
| 150414 | 27.31 | 120.42 | 78.01 | 75.99 | 2.14 | 1.51 | 1.48 | short-chain dehydrogenase reductase sdr |
| 165326 | 4.73 | 598.46 | 79.31 | 104.95 | 6.98 | 4.07 | 4.47 | mfs general substrate transporter |
| 153301 | 33.96 | 448.97 | 119.85 | 143.91 | 3.72 | 1.82 | 2.08 | l-aminoadipate-semialdehyde dehydrogenase |
| 172978 | 6.43 | 1058.62 | 23.05 | 20.70 | 7.36 | 1.84 | 1.69 | glycoside hydrolase family 43 protein |
|  | 24.69 | 123.86 | 70.53 | 64.22 | 2.33 | 1.51 | 1.38 | nuclear protein |
|  | 24.69 | 123.86 | 70.53 | 64.22 | 2.33 | 1.51 | 1.38 | hypothetical protein HETIRDRAFT_477710 |
| 156676 | 25.87 | 141.84 | 100.32 | 74.09 | 2.46 | 1.96 | 1.52 | opt oligopeptide transporter |
| 109187 | 3.17 | 93.55 | 22.10 | 12.40 | 4.88 | 2.80 | 1.97 | glycoside hydrolase family 18 protein |
| 388390 | 56.53 | 118.99 | 146.38 | 138.32 | 1.07 | 1.37 | 1.29 | oxysterol-binding protein |
| 65736 | 2.52 | 57.11 | 10.23 | 16.71 | 4.50 | 2.02 | 2.73 | glycoside hydrolase family 16 protein |
| 105792 | 11.49 | 60.74 | 39.07 | 37.29 | 2.40 | 1.77 | 1.70 | hypothetical protein HETIRDRAFT_105792 |
| 37838 | 14.88 | 587.31 | 118.68 | 138.71 | 5.30 | 3.00 | 3.22 | general substrate transporter |
| 460931 | 19.97 | 41.81 | 64.17 | 49.94 | 1.07 | 1.68 | 1.32 | phospholipase d |
| 421241 | 6.76 | 26.61 | 21.17 | 20.73 | 1.98 | 1.65 | 1.62 | wd40 repeat-like protein |
| 454451 | 3.64 | 60.86 | 14.97 | 16.61 | 4.06 | 2.04 | 2.19 | aromatic compound dioxygenase |
| 30910 | 3.36 | 26.94 | 16.89 | 18.48 | 3.00 | 2.33 | 2.46 | hypothetical protein HETIRDRAFT_30910 |
| 41624 | 7.57 | 101.66 | 32.59 | 35.11 | 3.75 | 2.11 | 2.21 | mfs general substrate transporter |
| 127023 | 8.60 | 40.16 | 50.89 | 42.73 | 2.22 | 2.56 | 2.31 | alpha beta-hydrolase |
| 10330 | 10.23 | 41.43 | 20.87 | 20.68 | 2.02 | 1.03 | 1.02 | glycoside hydrolase family 78 protein |
| 124437 | 6.57 | 105.01 | 53.30 | 85.04 | 4.00 | 3.02 | 3.69 | Glycoside Hydrolase Family 3 protein |
|  | 18.11 | 51.58 | 44.34 | 47.76 | 1.51 | 1.29 | 1.40 | 3-hydroxyacyl- dehydrogenase |
| 148374 | 5.35 | 92.37 | 12.51 | 20.80 | 4.11 | 1.23 | 1.96 | general substrate transporter |
| 64560 | 15.73 | 119.12 | 46.57 | 34.18 | 2.92 | 1.57 | 1.12 | subtilisin-like protein |
| 155702 | 9.66 | 107.15 | 140.41 | 124.21 | 3.47 | 3.86 | 3.68 | ncs1 nucleoside transporter family |
| 422795 | 2.75 | 10.80 | 8.30 | 10.53 | 1.98 | 1.60 | 1.94 | beta-glucosidase |
| 54043 | 3.96 | 13.17 | 14.06 | 14.52 | 1.73 | 1.83 | 1.87 | nad binding rossmann fold oxidoreductase |
| 456514 | 1.64 | 677.28 | 622.49 | 667.66 | 8.69 | 8.57 | 8.67 | hypothetical protein HETIRDRAFT_456514 |
|  | 4.56 | 18.77 | 33.37 | 30.98 | 2.04 | 2.87 | 2.76 | barwin-like endoglucanase |
| 477676 | 9.83 | 31.75 | 20.24 | 24.05 | 1.69 | 1.04 | 1.29 | hypothetical protein HETIRDRAFT_410566 |
| 171958 | 3.69 | 30.15 | 14.89 | 27.86 | 3.03 | 2.01 | 2.92 | hypothetical protein HETIRDRAFT_171958 |
| 471084 | 2.75 | 29.88 | 22.03 | 12.06 | 3.44 | 3.00 | 2.13 | amine oxidase |
| 106721 | 16.40 | 44.38 | 47.00 | 53.84 | 1.44 | 1.52 | 1.71 | hypothetical protein HETIRDRAFT_106721 |
| 52003 | 13.63 | 31.42 | 53.70 | 43.28 | 1.21 | 1.98 | 1.67 | amino acid transporter |
| 474665 | 40.40 | 85.22 | 87.52 | 75.60 | 1.08 | 1.12 | 0.90 | duf1752-domain-containing partial |
| 53130 | 14.52 | 29.73 | 44.26 | 36.61 | 1.03 | 1.61 | 1.33 | metallo peptidase m28 |
| 108567 | 6.64 | 14.48 | 17.50 | 16.79 | 1.12 | 1.40 | 1.34 | hypothetical protein HETIRDRAFT_108567 |
| 434979 | 3.38 | 9.36 | 31.58 | 57.23 | 1.47 | 3.22 | 4.08 | plp-dependent transferase |
| 316297 | 30.73 | 123.87 | 95.57 | 104.35 | 2.01 | 1.64 | 1.76 | hypothetical protein HETIRDRAFT_316297 |
| 329599 | 0.54 | 14.27 | 7.63 | 10.58 | 4.73 | 3.82 | 4.29 | xylitol dehydrogenase |

Table s8. List of the KEGG pathways mapped by CUGs specific to each species.

| *H. irregulare* | No. of genes | *H. occidentale* | No. of genes |
| --- | --- | --- | --- |
| 00010 Glycolysis / Gluconeogenesis | 4 | 00010 Glycolysis / Gluconeogenesis | 2 |
| 00020 Citrate cycle (TCA cycle) | 2 | 00030 Pentose phosphate pathway | 1 |
| 00030 Pentose phosphate pathway | 2 | 00040 Pentose and glucuronate interconversions | 5 |
| 00040 Pentose and glucuronate interconversions | 2 | 00051 Fructose and mannose metabolism | 3 |
| 00051 Fructose and mannose metabolism | 1 | 00053 Ascorbate and aldarate metabolism | 1 |
| 00052 Galactose metabolism | 1 | 00061 Fatty acid biosynthesis | 1 |
| 00053 Ascorbate and aldarate metabolism | 1 | 00062 Fatty acid elongation | 1 |
| 00061 Fatty acid biosynthesis | 1 | 00071 Fatty acid degradation | 3 |
| 00071 Fatty acid degradation | 4 | 00240 Pyrimidine metabolism | 2 |
| 00072 Synthesis and degradation of ketone bodies | 1 | 00260 Glycine, serine and threonine metabolism | 2 |
| 00100 Steroid biosynthesis | 1 | 00280 Valine, leucine and isoleucine degradation | 3 |
| 00130 Ubiquinone and other terpenoid-quinone biosynthesis | 1 | 00290 Valine, leucine and isoleucine biosynthesis | 1 |
| 00190 Oxidative phosphorylation | 2 | 00310 Lysine degradation | 1 |
| 00220 Arginine biosynthesis | 2 | 00330 Arginine and proline metabolism | 1 |
| 00230 Purine metabolism | 1 | 00350 Tyrosine metabolism | 1 |
| 00240 Pyrimidine metabolism | 1 | 00380 Tryptophan metabolism | 3 |
| 00250 Alanine, aspartate and glutamate metabolism | 1 | 00410 beta-Alanine metabolism | 2 |
| 00260 Glycine, serine and threonine metabolism | 4 | 00460 Cyanoamino acid metabolism | 2 |
| 00261 Monobactam biosynthesis | 1 | 00500 Starch and sucrose metabolism | 4 |
| 00270 Cysteine and methionine metabolism | 3 | 00510 N-Glycan biosynthesis | 1 |
| 00280 Valine, leucine and isoleucine degradation | 3 | 00511 Other glycan degradation | 2 |
| 00300 Lysine biosynthesis | 1 | 00513 Various types of N-glycan biosynthesis | 1 |
| 00310 Lysine degradation | 2 | 00520 Amino sugar and nucleotide sugar metabolism | 3 |
| 00330 Arginine and proline metabolism | 3 | 00521 Streptomycin biosynthesis | 1 |
| 00340 Histidine metabolism | 2 | 00531 Glycosaminoglycan degradation | 1 |
| 00350 Tyrosine metabolism | 3 | 00561 Glycerolipid metabolism | 4 |
| 00360 Phenylalanine metabolism | 4 | 00562 Inositol phosphate metabolism | 2 |
| 00361 Chlorocyclohexane and chlorobenzene degradation | 1 | 00564 Glycerophospholipid metabolism | 1 |
| 00362 Benzoate degradation | 1 | 00565 Ether lipid metabolism | 1 |
| 00380 Tryptophan metabolism | 4 | 00592 alpha-Linolenic acid metabolism | 1 |
| 00400 Phenylalanine, tyrosine and tryptophan biosynthesis | 2 | 00603 Glycosphingolipid biosynthesis - globo series | 1 |
| 00401 Novobiocin biosynthesis | 1 | 00604 Glycosphingolipid biosynthesis - ganglio series | 1 |
| 00410 beta-Alanine metabolism | 3 | 00627 Aminobenzoate degradation | 3 |
| 00440 Phosphonate and phosphinate metabolism | 1 | 00640 Propanoate metabolism | 1 |
| 00450 Selenocompound metabolism | 1 | 00643 Styrene degradation | 1 |
| 00460 Cyanoamino acid metabolism | 1 | 00650 Butanoate metabolism | 1 |
| 00514 Other types of O-glycan biosynthesis | 1 | 00670 One carbon pool by folate | 1 |
| 00520 Amino sugar and nucleotide sugar metabolism | 3 | 00680 Methane metabolism | 4 |
| 00561 Glycerolipid metabolism | 2 | 00710 Carbon fixation in photosynthetic organisms | 1 |
| 00564 Glycerophospholipid metabolism | 4 | 00740 Riboflavin metabolism | 1 |
| 00565 Ether lipid metabolism | 1 | 00770 Pantothenate and CoA biosynthesis | 1 |
| 00592 alpha-Linolenic acid metabolism | 1 | 00780 Biotin metabolism | 2 |
| 00600 Sphingolipid metabolism | 1 | 00910 Nitrogen metabolism | 1 |
| 00620 Pyruvate metabolism | 4 | 00930 Caprolactam degradation | 1 |
| 00621 Dioxin degradation | 1 | 00940 Phenylpropanoid biosynthesis | 1 |
| 00623 Toluene degradation | 1 | 00950 Isoquinoline alkaloid biosynthesis | 1 |
| 00624 Polycyclic aromatic hydrocarbon degradation | 1 | 00965 Betalain biosynthesis | 1 |
| 00625 Chloroalkane and chloroalkene degradation | 2 | 01040 Biosynthesis of unsaturated fatty acids | 2 |
| 00626 Naphthalene degradation | 2 | 01200 Carbon metabolism | 5 |
| 00627 Aminobenzoate degradation | 1 | 01210 2-Oxocarboxylic acid metabolism | 1 |
| 00630 Glyoxylate and dicarboxylate metabolism | 7 | 01212 Fatty acid metabolism | 3 |
| 00650 Butanoate metabolism | 2 | 01230 Biosynthesis of amino acids | 3 |
| 00670 One carbon pool by folate | 2 | 02010 ABC transporters | 1 |
| 00680 Methane metabolism | 4 | 03030 DNA replication | 1 |
| 00710 Carbon fixation in photosynthetic organisms | 4 | 03320 PPAR signaling pathway | 2 |
| 00770 Pantothenate and CoA biosynthesis | 1 | 03430 Mismatch repair | 1 |
| 00780 Biotin metabolism | 1 | 03450 Non-homologous end-joining | 1 |
| 00790 Folate biosynthesis | 1 | 04014 Ras signaling pathway (1) | 1 |
| 00830 Retinol metabolism | 1 | 04024 cAMP signaling pathway | 2 |
| 00900 Terpenoid backbone biosynthesis | 1 | 04068 FoxO signaling pathway | 1 |
| 00903 Limonene and pinene degradation | 1 | 04071 Sphingolipid signaling pathway | 1 |
| 00910 Nitrogen metabolism | 1 | 04110 Cell cycle | 3 |
| 00920 Sulfur metabolism | 1 | 04111 Cell cycle - yeast | 5 |
| 00940 Phenylpropanoid biosynthesis | 1 | 04113 Meiosis - yeast | 2 |
| 00950 Isoquinoline alkaloid biosynthesis | 1 | 04114 Oocyte meiosis | 3 |
| 00960 Tropane, piperidine and pyridine alkaloid biosynthesis | 1 | 04120 Ubiquitin mediated proteolysis | 1 |
| 00980 Metabolism of xenobiotics by cytochrome P450 | 3 | 04141 Protein processing in endoplasmic reticulum | 1 |
| 00982 Drug metabolism - cytochrome P450 | 2 | 04142 Lysosome | 4 |
| 00983 Drug metabolism - other enzymes | 1 | 04144 Endocytosis | 2 |
| 01040 Biosynthesis of unsaturated fatty acids | 2 | 04146 Peroxisome | 3 |
| 01051 Biosynthesis of ansamycins | 1 | 04152 AMPK signaling pathway | 1 |
| 01200 Carbon metabolism | 11 | 04614 Renin-angiotensin system | 1 |
| 01210 2-Oxocarboxylic acid metabolism | 2 | 04622 RIG-I-like receptor signaling pathway | 1 |
| 01212 Fatty acid metabolism | 2 | 04626 Plant-pathogen interaction | 1 |
| 01220 Degradation of aromatic compounds | 2 | 04666 Fc gamma R-mediated phagocytosis | 1 |
| 01230 Biosynthesis of amino acids | 7 | 04724 Glutamatergic synapse | 1 |
| 02020 Two-component system | 1 | 04912 GnRH signaling pathway | 1 |
| 03050 Proteasome | 1 | 04914 Progesterone-mediated oocyte maturation | 2 |
| 03320 PPAR signaling pathway | 1 | 04916 Melanogenesis | 1 |
| 04011 MAPK signaling pathway - yeast | 1 | 04962 Vasopressin-regulated water reabsorption | 1 |
| 04071 Sphingolipid signaling pathway | 1 | 05010 Alzheimer's disease | 1 |
| 04111 Cell cycle - yeast | 1 | 05166 HTLV-I infection | 1 |
| 04113 Meiosis - yeast | 1 | 05203 Viral carcinogenesis | 1 |
| 04142 Lysosome | 1 | 05206 MicroRNAs in cancer | 1 |
| 04145 Phagosome | 1 | 05231 Choline metabolism in cancer | 1 |
| 04146 Peroxisome | 5 |  |  |
| 04152 AMPK signaling pathway | 2 |  |  |
| 04210 Apoptosis | 1 |  |  |
| 04721 Synaptic vesicle cycle | 1 |  |  |
| 04724 Glutamatergic synapse | 1 |  |  |
| 04727 GABAergic synapse | 1 |  |  |
| 04910 Insulin signaling pathway | 2 |  |  |
| 04920 Adipocytokine signaling pathway | 1 |  |  |
| 04932 Non-alcoholic fatty liver disease (NAFLD) | 1 |  |  |
| 04966 Collecting duct acid secretion | 1 |  |  |
| 04976 Bile secretion | 1 |  |  |
| 05110 Vibrio cholerae infection | 1 |  |  |
| 05120 Epithelial cell signaling in Helicobacter pylori infection | 1 |  |  |
| 05143 African trypanosomiasis | 1 |  |  |
| 05146 Amoebiasis | 1 |  |  |
| 05204 Chemical carcinogenesis | 3 |  |  |
| 05323 Rheumatoid arthritis | 1 |  |  |
